# Sex differences in the risk of autistic-related traits in toddlers born to mothers with perinatal depression: Evidence from human cohort and mouse study

**DOI:** 10.1038/s41380-026-03456-z

**Published:** 2026-02-04

**Authors:** Changrong Duan, Zhiqian Yu, Xue Li, Mai Sakai, Yuko Maejima, Kenju Shimomura, Tomoyuki Furuyashiki, Saya Kikuchi, Natsuko Kobayashi, Kazuto Sasaki, Tasuku Matsuki, Hiroshi Komatsu, Mizuki Hino, Yasuto Kunii, Tomoko Kasahara, Mami Ishikuro, Keiko Murakami, Masatsugu Orui, Takaaki Abe, Fuji Nagami, Nobuo Fuse, Soichi Ogishima, Kengo Kinoshita, Masayuki Yamamoto, Naoki Nakaya, Atsushi Hozawa, Taku Obara, Shinichi Kuriyama, Hiroaki Tomita

**Affiliations:** 1 Department of Psychiatry, Graduate School of Medicine, Tohoku University, Sendai, 980-8573 Japan; 2 Tohoku Medical Megabank Organization, Tohoku University, Sendai, 980-8573 Japan; 3 Department of Regional Alliance for Promoting Liaison Psychiatry, Graduate School of Medicine, Tohoku University, Sendai, 980-8573 Japan; 4 Department of Psychiatric Nursing, Graduate School of Medicine, Tohoku University, Sendai, 980-8575 Japan; 5 Department of Bioregulation and Pharmacological Medicine, Fukushima Medical University School of Medicine, Fukushima, 960-1295 Japan; 6 Department of Obesity and Inflammation Research, Fukushima Medical University School of Medicine, Fukushima, 960-1295 Japan; 7 Department of Pharmacology, Graduate School of Medical and Dental Sciences, Institute of Science Tokyo, Tokyo, 113-8510 Japan; 8 Department of Psychiatry, Tohoku University Hospital, Sendai, 980-8573 Japan; 9 Department of Disaster Psychiatry, International Research Institute for Disaster Science, Tohoku University, Sendai, 980-8573 Japan; 10 Department of Clinical Biology and Hormonal Regulation, Graduate School of Medicine, Tohoku University, Sendai, 980-8573 Japan; 11 Department of Health and Social Behavior, Graduate School of Medicine, The University of Tokyo, Tokyo, 113-0033 Japan; 12 Department of Biomedical Engineering Regenerative and Biomedical Engineering Medical Science, Graduate School of Biomedical Engineering, Tohoku University, Sendai, 980-8575 Japan; 13 Advanced Research Center for Innovations in Next-Generation Medicine, Tohoku University, Sendai, 980-8573 Japan; 14 Graduate School of Information Sciences, Tohoku University, Sendai, 980-8579 Japan; 15 Department of Public Health, Graduate School of Medicine, Tohoku University, Sendai, 980-8573 Japan; 16 Division of Disaster Public Health, International Research Institute for Disaster Science, Tohoku University, Sendai, 980-8573 Japan

**Keywords:** Autism spectrum disorders, Depression

## Abstract

Maternal perinatal depression (MPD) is associated with reduced maternal plasma oxytocin (OXT) levels and an increased risk of autism spectrum disorder (ASD) in offspring. Using data from 23,218 Japanese mother–child pairs, we evaluated the relationship between MPD—assessed with the Kessler Psychological Distress Scale (K6) and the Edinburgh Postnatal Depression Scale (EPDS)—and autistic-related traits (ART) in toddlers, measured by the Tokyo Autistic Behavior Scale (TABS). We also tested the potential causal relationship of maternal stress exposure on OXT, its receptor (OXTR), and offspring outcomes using a prenatal stress-exposed mouse model. In the human cohort study, higher K6 or EPDS scores during pregnancy and postpartum were significantly associated with increased TABS scores in toddlers. Offspring of mothers with MPD (K6 or EPDS score ≥ 9) during pregnancy or postpartum exhibited a higher risk of ART (TABS score ≥ 15; *P* < 0.05). This risk was particularly pronounced in female toddlers exposed to MPD during pregnancy and postpartum (ORs: 5.805–9.367; *P* < 0.05). Female toddlers born to mothers with MPD also had lower birth weight, and their ART were positively correlated with K6 scores during mid-gestation and with impaired maternal bonding postpartum. In the mouse model, chronically stressed dams displayed depressive-like behaviors, and their female juveniles exhibited increased self-grooming and impaired social interaction. Furthermore, *OXTR* mRNA levels were significantly reduced in the prefrontal cortex of female juveniles from stressed dams. These findings suggest that MPD increases the risk of ART, particularly in females, highlighting potential sex-specific mechanisms underlying ASD susceptibility.

## Introduction

Maternal perinatal depression (MPD), encompassing both antenatal depression and postpartum depression (PPD), affects approximately 10–20% of women during pregnancy and the postpartum period [1–3]. Women with antenatal depression are often at a higher risk of developing PPD after delivery [4]. For mothers with MPD, consistent risk factors include a history of depression, lower education levels, unintended pregnancy, inadequate social support, poor childcare quality, and relationship difficulties with partners [5–7], in addition to biological factors, including hormonal changes, inflammatory processes, and genetic predispositions, also contribute to the risk [8–10]. These social, communication, and behavioral factors may impact offspring neurodevelopment, contributing to an increased prevalence of autism spectrum disorders (ASD) in their children [11–13].

Several studies have indicated differences in the frequency and manner of expression of autistic phenotypes between males and females. A recent review reported that ASD prevalence is more than four times higher in males than in females and is often comorbid with other neurodevelopmental disorders, such as intellectual disability, epilepsy, and attention-deficit/hyperactivity disorder [14]. Moreover, an animal study in rats found that pre-gestational maternal stress resulted in depressive-like behaviors in adolescent offspring of both sexes, with increased anxiety-like behavior observed specifically in female offspring [15]. However, little attention has been given to MPD’s influence on the development of autistic traits in offspring, particularly with respect to sex differences.

While the mechanisms of MPD on offspring’s autistic phenotypes have garnered increasing attention, the molecular risk factors remain largely unexplored [16]. Several biological systems have been implicated, including neurotrophic factors such as brain-derived neurotrophic factor (BDNF) and its receptor, tropomyosin receptor kinase B (TrkB), which regulate stress responses and neuronal growth [17]. BDNF/TrkB signaling has been reported to be decreased in mothers with perinatal depression [18] but increased in children with ASD [19], indicating disruption of this pathway [20]. Another system of particular interest is oxytocin (OXT), which aids in childbirth, promotes milk ejection during breastfeeding, and promotes mother–infant bonding as a neuropeptide [21]. Skrundz et al. reported that lower plasma OXT concentrations in mid-pregnancy significantly predicted PPD symptoms at 2 weeks postpartum, suggesting that enhancing OXT release during pregnancy could be a potential target for PPD prevention [22] and for potentially mitigating its adverse effects on the mother–child relationship [23]. OXT has been investigated as a potentially effective agent for ASD treatment [24], but its limited benefits render its efficacy controversial [25]. Serum OXT levels show sex-specific associations with ASD-related symptoms but are not associated with the severity of intellectual disabilities [26]. Notably, sex differences in OXT systems—characterized in several studies by higher OXT levels in females and higher OXT receptor (OXTR) expression in males—may play a role in the sex-specific regulation of both healthy and impaired social behaviors [27]. Significantly lower levels of OXTR densities but not transcripts have been reported in substantia nigra in postmortem human brain from female patients with ASD [28]. Despite these findings, the expression of OXT and OXTR in the maternal and toddlers’ brains and its relationship to maternal perinatal stress exposure remain unclear.

OXT also influences the activity of various immune cells, contributing to the maintenance of immune homeostasis and the suppression of stress-associated immune disorders [29]. Additionally, clinical studies have consistently associated maternal immune activation during pregnancy with an increased risk of autism-related outcomes in offspring [30, 31]. Microglia, the brain’s primary immune cells, play a crucial role in neurodevelopment [32, 33] and are key contributors to pathological conditions in microangiopathic disorders [34]. In the context of ASD, microglial dysfunction is implicated in its pathogenesis, characterized by microglial activation, phenotypic shifts, and dysregulated synaptic pruning [35, 36]. Recently, we reported reduced plasma cytokines along with immune-associated metabolic disturbances in women with PPD [10, 37], as well as stress-induced alterations in microglial immune responses, including TNF-α in rodent models [38–40]. Furthermore, we reported that microglial OXT in both humans and mice positively correlates with the secretion of cytokines [41, 42], and sex differences in microglial *Oxt* transcript expression in mice and experimenters under acute stress [43]. Based on these findings, we hypothesize that stress during pregnancy disrupts OXT generation in the maternal brain, potentially dysregulating physiological functions [44], which may contribute to sex differences in ASD pathogenesis in offspring. This study evaluated the relationship between MPD in mothers and autistic-related traits (ART) in their toddlers, as well as its potential contribution to sex differences in these clinical characteristics. Data from a Japanese cohort study were analyzed and supplemented by a mouse model of MPD designed to validate results of cohort. Furthermore, in the murine model, the potential molecular mechanisms through which prenatal stress influences OXT expression in the hypothalamic supraoptic nucleus (SON), paraventricular nucleus (PVN) neurons, and microglia in dams, as well as the sex differences in the transcription of *Oxt* and *Oxtr* in the prefrontal cortex (PFC) of juveniles were evaluated.

## Materials and methods

### Human cohort study and protocol for assessing depressive symptoms and autistic behaviors

This analysis targeted women during pregnancy and childbirth from the Tohoku Medical Megabank Project (TMM) Birth and Three-Generation cohort (TMM BirThree Cohort Study; the Ethics Committee of the Tohoku University Graduate School of Medicine, Approval No. *2024-1-677*), a general population-based prospective cohort study that began in 2011. Women diagnosed by gestational diabetes mellitus was excluded.

The mental psychological distress of mothers was evaluated using scores from the Japanese version of the Kessler Psychological Distress Scale (K6), which consisted of six items and was collected at two time points: early (0–15 weeks) and mid (16–27 weeks) gestation. Furthermore, the Japanese version of the K6 comprises six validated questions. The total K6 score, ranging from 0 to 24, is commonly categorized into four groups: normal (0–5), low (6–8), moderate (9–13), and high (14–24) [37]. In the current study, K6 scores ≥9 was used to indicate maternal psychological distress (PD) during gestation [2]. The Japanese version of the Edinburgh Postnatal Depression Scale (EPDS), which consists of 10 items, was collected at the 4–5 weeks postpartum. Women with EPDS scores of 9 or higher ( ≥ 9) [37] were assigned to the PPD group, while those with scores below 9 (<9) were designated as controls. The Japanese version of the Mother-to-Infant Bonding Scale (MIBS) was used to assess bonding failure one month after delivery, with higher scores indicating greater severity of bonding failure associated with maternal psychological distress [45]. Data on mothers and their children used in this study were collected and processed until March 14, 2025. Maternal psychiatric history (including major depression, anxiety disorder, ADHD, ASD, bipolar disorder, schizophrenia, and PTSD), smoking and drinking habits, educational attainment, household income, use of antipsychotic medications (depression and anxiety), selective serotonin reuptake inhibitors (SSRIs: escitalopram, sertraline, fluvoxamine), other antidepressants (mirtazapine), hypnotics (zolpidem, suvorexant), and benzodiazepines or related anxiolytics/hypnotics (ethyl loflazepate, etizolam, flunitrazepam, clotiazepam, lorazepam) were also analyzed as covariates. Since fluoxetine carries a high risk of inducing ASD [46] and is not a prescribed medication in Japan, none of the mothers and toddlers in the current cohort were exposed to this antidepressant. We obtained written informed consent from all participants who agreed to participate in the TMM BirThree Cohort Study (RHQ No. *2013-1-103-1, 2021-1-266*) [37]. This report was conducted according to the STROBE guidelines for reporting observational studies in epidemiology [47].

The Tokyo Autistic Behavior Scale (TABS) [48], consisting of 39 items, including four areas: interpersonal-social relationship, language-communication, habit-mannerism, and others, were used to assess autistic-related characteristics in 2–3-years-old toddlers [49]. Moreover, we used a cut-off score of ≥15 on the TABS for diagnosing ART in toddlers [49]. The current analyses include TABS data collected from one child, randomly selected in the case of multiple births (e.g., twins). The Mann-Whitney *U* test and Spearman rank correlation test were used to compare different groups. The significance of incidence rates between the two groups was evaluated using Chi-squared test with MedCalc^®^ Statistical Software version 23.0.6 (MedCalc Software Ltd, Ostend, Belgium; https://www.medcalc.org; 2024).

A total of 23,218 women with K6 or EPDS scores and their toddlers with TABS scores (male: n = 11,978; female: n = 11,240) were analyzed. Finally, the relationships between maternal mental status, including PD, PPD, and bonding, and their toddlers’ ART were evaluated using maternal K6, EPDS, and MIBS scores, along with TABS scores of their live singleton-born toddlers or those randomly selected from multiple births, at three time points: early gestation, mid-gestation, and postpartum.

### Animals

All experimental procedures were reviewed and approved by the Institutional Animal Care and Use Committee (IACUC) of the Tohoku University Environmental & Safety Committee (approval number: *2023MdA-023-03*). This study used 8-week-old sexually experienced male C57BL/6 J mice and microglia-specific GFP-expressing CX3CR1^GFP/+^ virgin female mice. CX3CR1^GFP/GFP^ mice were obtained from the Jackson Laboratory and crossed with C57BL/6 J mice, as previously described [50]. Male C57BL/6 J mice were purchased from SLC Japan Inc. and acclimated to the laboratory environment for one week before the start of the experiment. Upon arrival, animals were housed in groups of three under standardized conditions as described below.

### General procedure

**Fig. 1:**
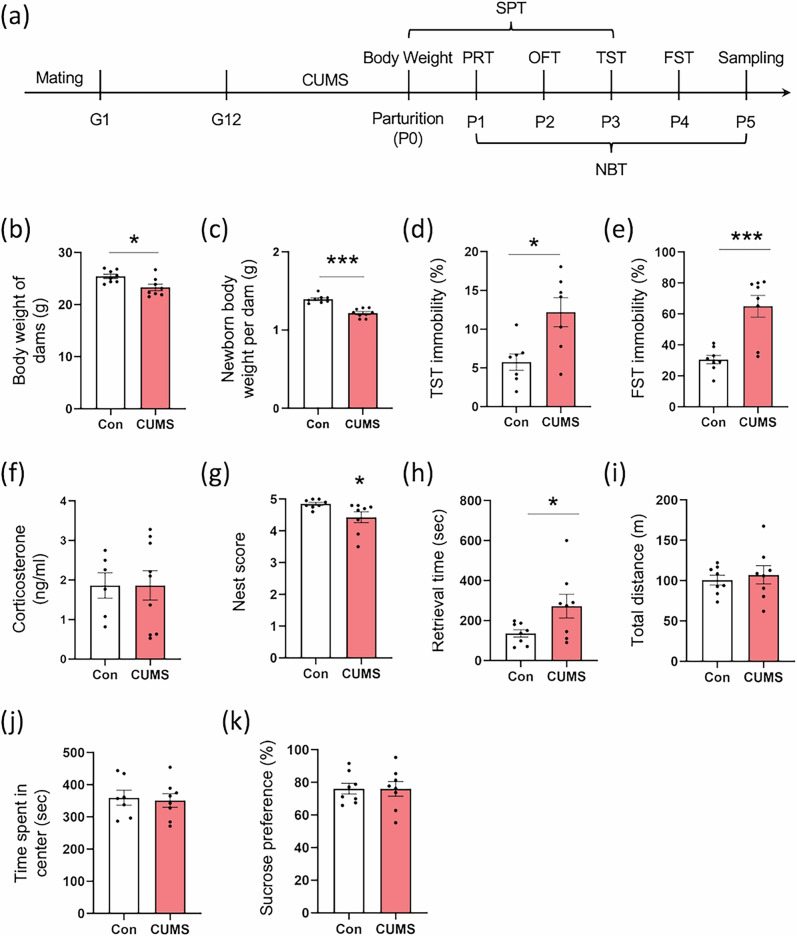
The effects of prenatal stress on body weight and depressive-like behaviors of dams. **a** Schedule of behavioral experiments. CUMS is performed on gestational day 12 (G12) until the day of parturition, which was designated as postnatal day (P0). Behavioral experiments accessed from P1 to P5. The average body weight of dams (**b**) and newborns per dam (**c**) were measured. **d** and **e** The results of the tail suspension test and forced swimming test are shown using the percentage time of immobility. **f** Serum corticosterone in dams on P5. **g** The score of the nest building test. **h** The time dams retrieved all the pups in the pup retrieval test. **i** and **j** The open-field test analyzed the total traveling distance and time spent in the center zone. **k** The percentage of sucrose solution taken in the sucrose preference test. Behavior test, Control, n = 8; CUMS, n = 8. Serum corticosterone, Control, n = 7; CUMS, n = 9. G, gestation; P, postpartum day; Control, non-stressed dams; CUMS, chronic unpredictable mild stress exposed dams; OFT, open-field test; SPT, sucrose preference test; TST, tail suspension test; FST, forced swim test; PRT, pup retrieval test; NBT, nest building test. Bar graphs are presented as mean ± SEM. *P < 0.05, ***P < 0.001. vs. Control, Student’s *t*-test.

On arrival, animals were housed in groups of three in standard polycarbonate cages (182 × 260 × 128 mm; CL-0103–1 CLEA Japan, Inc., Shizuoka, Japan) in the animal housing facility of the Tohoku University Graduate School of Medicine under controlled laboratory conditions (12-h light/dark cycle, lights on at 9:00 AM; temperature 24 ± 2 °C; relative humidity 50 ± 10%), with food and water available *ad libitum*. Female CX3CR1^GFP/GFP^ mice were mated with 8-week-old C57BL/6 J male mice. Each paired female and male mice were housed in a cage. After day 2, female mice with copulatory plugs were placed into a separate home cage while male mice were removed; this day was designated as gestational day (G1), at which time the subjects were randomly assigned to the stress or control group. All mice were housed individually in cages and provided with sawdust bedding. The chronic unpredictable mild stress (CUMS) procedure was initiated from day G12 until childbirth (postpartum day (P0) (Fig. 1a). After CUMS exposure, half of the dams were used in behavior tests and euthanized for tissue collection. The other half of the dams continued to nurture the pups for three weeks after weaning. During the stress regimen processing, CUMS-exposed dams were housed in a specific room without contact with their controls. Details of mice used for breeding, real-time (RT)-qPCR, in situ hybridization, and behavioral tests were summarized in Supplementary methods and Fig. S1.

### CUMS procedure

The CUMS procedure was referenced and modified from a previous report [51]. The stress regimen consisted of ten unpredictable mild stressors for different periods, as summarized in Table S1. From day G12 to childbirth (P0) (9―11 days), the following stress were randomly exposed to pregnant dams (Fig. 1a): restraint stress (2 h), restricted access to food (6 h), forced swimming (10 min), continuing light overnight, foreign object in a cage (16 h), cage tilt 30° from the horizontal (7 h), white noise (80 dB, 3 h), wet bed (30 mL water at the middle of sawdust bedding in the cage; 16 h), paired housing with stressed pregnant mice (16 h), and confinement to small cages (mice were moved to a cage measuring 20 × 13 ×14 cm; 16 h).

### Statistical analysis

#### Cohort study

Variables (K6, EPDS, TABS, and MIBS scores) were analyzed as continuous measures (e.g., correlations), and the results were consistent with those obtained using categorical cutoffs. A categorical approach (e.g., K6 or EPDS scores) was also applied, as these thresholds have recognized clinical relevance and enable comparison with our previous studies [2, 10, 37]. Univariate Ordinary Least Squares (OLS) linear regression models were performed with maternal distress as a binary exposure variable (K6 or EPDS < 9 vs. ≥ 9), adjusting for maternal psychiatric history, smoking, drinking, education, household income, and antipsychotic use, with an interaction term between maternal distress and child sex to examine potential sex-specific effects on TABS scores. Group differences were also examined using nonparametric Mann–Whitney *U* tests to account for unequal sample sizes between groups.

#### Animal experiments

Behavioral test, RT-qPCR, ELISA, and in situ outcomes of dams were analyzed using Student’s *t*-test between CUMS-dams and controls. Newborn weight, behavioral measures, and RT-qPCR outcomes in juveniles were analyzed for the effects of maternal stress (CUMS vs. control), litters, sex (male vs. female), and their interaction. All analyses were performed in Python (statsmodels package, version 0.14.0) or GraphPad Prism version 10 (GraphPad Software, Inc., San Diego, CA, USA). Two-sided *P* values < 0.05 were considered statistically significant. Data are presented as mean ± SEM.

## Results

### The impact of perinatal depression on the development of autistic traits in toddlers

We first examined the correlations among K6, EPDS, TABS, and MIBS. As shown in Table S2, K6 scores during early pregnancy and mid-gestation were positively correlated with EPDS scores. Furthermore, higher K6 and EPDS scores were positively correlated with higher TABS and MIBS scores, and TABS and MIBS were also positively correlated with each other, suggesting that more severe maternal psychological distress is associated with poorer mother–infant bonding and more pronounced autistic traits in offspring. Based on the TMM BirThree Cohort Study, maternal PD prevalence, defined as K6 ≥ 9, was 14.59% (n = 1,665) during early gestation and 10.07% (n = 1,145) during mid-gestation (Table 1). Mothers with early or mid-PD were significantly younger compared to those without PD (*P* < 0.0001, Table 1). A significant positive correlation was observed between early and mid-gestational K6 scores (R = 0.663, *P* < 0.001) (Table 1). Furthermore, maternal PPD was identified one month after delivery, with 13.78% (n = 3,200) of mothers meeting the threshold for EPDS ≥ 9 (Table 1). Further analyses were conducted using data from paired mother–toddler dyads across three independent groups: early gestation, mid-gestation, and postpartum.

**Table 1:** Association between maternal mental status and toddlers’ autistic-related traits.

	Mothers (n)		K6 or EPDS Socre	Early gestation (K6) (11,410)	Mid-gestation (K6) (11,362)	Postpartum (EPDS) (23,218)
**Mothers**	PD/PPD	(n) (age, MEAN ± SD)	< 9	9745 (31.83 ± 4.72)	10,217 (31.81 ± 4.73)	20,018 (31.22 ± 4.72)
			≥ 9	1665 (30.55 ± 4.93^###^)	1145 (30.24 ± 4.90^###^)	3200 (29.83 ± 4.64^###^)
		(% of ≥ 9)		14.59	10.07	13.78
	Body weight during mid pregnancy (n) (kg, MEAN ± SD)		< 9	7585 (63.83 ± 11.16)	7977 (63.79 ± 11.04)	19,043 (63.52 ± 10.06)
			≥ 9	1320 (63.61 ± 9.35)	892 (64.01 ± 9.80)	3442 (62.39 ± 9.22)
	Body weight after delivery (n) (kg, MEAN ± SD)		< 9	9153 (56.81 ± 10.04)	9596 (56.80 ± 9.97)	17,765 (56.87 ± 10.08)
			≥ 9	1539 (56.63 ± 8.69)	1055 (56.70 ± 8.84)	2790 (56.70 ± 8.71)
	PD/PPD	Early pregnancy (n) (K6, MEAN ± SD)	< 9 ≥ 9	9745 (2.57 ± 2.44) 1665 (12.72 ± 3.82)	9681 (2.57 ± 2.44) 1653 (12.72 ± 3.81^@@@^)	9745 (2.57 ± 2.44) 1665 (12.72 ± 3.82^@@@^)
		Mid pregnancy (n) (K6, MEAN ± SD)	< 9 ≥ 9	10,192 (2.41 ± 2.36) 1142 (12.09 ± 3.25^@@@^)	10,217 (2.41 ± 2.36) 1145 (12.10 ± 3.26)	10,217 (2.41 ± 2.36) 1145 (12.10 ± 3.26^@@@^)
		Postpartum 1 mo. (n) EPDS, MEAN ± SD	< 9 ≥ 9	9854 (3.93 ± 2.22) 1556 (11.65 ± 3.16^@@@^)	9816 (3.93 ± 2.22) 1546 (11.65 ± 3.16^@@@^)	20,018, (3.98 ± 2.22) 3200 (11.67 ± 3.21)
				Early K6	Mid-K6	EPDS
	Correlations R (P value)	Early K6		1	0.663 (<0.001)	0.427 (<0.001)
		Mid-K6		0.663 (<0.001)	1	0.473 (<0.001)
		EPDS		0.427 (<0.001)	0.473 (<0.001)	1
		TABS		0.143 (<0.001)	0.152 (<0.001)	0.180 (<0.001)
**Toddlers**	**Toddlers (n)**			**11,410**	**11,362**	**23,218**
	Birth weight of newborn (n) (kg, MEAN ± SD)		< 9	9472 (3.02 ± 0.43)	9942 (3.02 ± 0.43)	18,661 (3.01 ± 0.43)
			≥ 9	1629 (3.00 ± 0.43)	1113 (2.99 ± 0.42)*	2977 (2.99 ± 0.53)*
	PD/PPD (n) (TABS, MEAN ± SD)		< 9	9745 (3.88 ± 2.88)	10,217 (3.92 ± 2.91)	20,018 (4.05 ± 2.95)
			≥ 9	1665 (4.80 ± 3.20***)	1145 (4.83 ± 3.16***)	3200 (4.91 ± 3.22***)
	PD/PPD & TABS Correlation (R, P-value)	Male& female	< 9	0.105 (<0.0001)	0.135 (<0.0001)	0.130 (<0.0001)
			≥ 9	0.060 (<0.05)	0.080 (<0.01)	0.131 (<0.0001)
		(male)	< 9	0.108 (<0.0001)	0.131 (<0.0001)	0.130 (<0.0001)
			≥ 9	0.058 (n.s)	0.057 (n.s)	0.107 (<0.0001)
		(female)	< 9	0.106 (<0.0001)	0.141 (<0.0001)	0.126 (<0.0001)
			≥ 9	0.044 (n.s)	0.102 (<0.05)	0.146 (<0.0001)
	Sex differences of TABS	Male (n) (TABS, MEAN ± SD)	< 9	5034 (4.10 ± 2.91)	5240 (4.15 ± 2.95)	10,303 (4.26 ± 2.98)
			≥ 9	851 (5.04 ± 3.23)	611 (4.95 ± 3.08)	1675 (5.09 ± 3.17)
		Female (n) (TABS, MEAN ± SD)	< 9	4711 (3.64 ± 2.84^$$$^)	4977 (3.68 ± 2.84^$$$^)	9715 (3.80 ± 2.89^$$$^)
			≥ 9	814 (4.54 ± 3.15^& & &^)	534 (4.69 ± 3.25^& & &^)	1525 (4.71 ± 3.27^& & &^)
	PD/PPD ART (n) (TABS ≥ 15)	Male (n/total)	< 9	7/5034	9/5240	23/10,303
			≥ 9	3/851	1/611	8/1675
		Female (n/total)	< 9	3/4711	3/4977	5/9715
			≥ 9	3/814^£^	3/534^£^	6/1525^£^
	PD/PPD (n) (MIBS, MEAN ± SD)		< 9	9477 (2.13 ± 2.29)	9946 (2.16 ± 2.31)	18,665 (2.24 ± 2.34)
			≥ 9	1631 (3.28 ± 2.99***)	1115 (3.60 ± 3.18***)	2981 (3.38 ± 3.08***)
	PD/PPD MIBS&TABS Correlation (R, P-value)	Male& female	< 9	0.173 (<0.0001)	0.210 (<0.0001)	0.167 (<0.0001)
			≥ 9	0.101 (<0.0001)	0.106 (<0.001)	0.100 (<0.0001)
		(male)	< 9	0.137 (<0.0001)	0.140 (<0.0001)	0.113 (<0.0001)
			≥ 9	0.114 (<0.001)	0.104 (<0.05)	0.050 (n.s.)
		(female)	< 9	0.151 (<0.0001)	0.144 (<0.0001)	0.125 (<0.0001)
			≥ 9	0.126 (<0.001)	0.160 (<0.001)	0.107 (<0.0001)

*PD* maternal perinatal psychological distress, *PPD* maternal postpartum depression, *ART* autistic-related traits, *K6* Japanese version of the Kessler Psychological Distress Scale, *EPDS* Japanese version of the Edinburgh Postnatal Depression Scale, *TABS* Tokyo Autistic Behavior Scale, *MIBS* Mother-to-Infant Bonding Scale. ###, vs. maternal age in the < 9 group (K6 < 9 or EPDS < 9), *P* < 0.0001; @@@, vs. K6 scores in the < 9 group (EPDS < 9), *P* < 0.0001; *, vs. newborn weights in the <9 group (K6 <9 or EPDS <9), *P* < 0.05; ***, vs. TABS scores in the < 9 group (K6 < 9 or EPDS < 9), *P* < 0.0001; $$$, vs. male TABS scores in the < 9 group (K6 < 9 or EPDS < 9), *P* < 0.0001; & & &, vs. male TABS scores in the ≥ 9 group (K6 ≥ 9 or EPDS ≥ 9), *P* < 0.0001; £, vs. ART scores of female toddlers born to mothers without PD or PPD (K6 < 9 or EPDS < 9), *P* < 0.05.

No significant differences in maternal body weight were observed between mothers with early-PD, mid-PD, or PPD and their respective control groups during pregnancy and postpartum (*P* > 0.05, Table 1). Additionally, there were no sex differences in the number of newborns, nor any effect of PD on the number of stillbirths, neonatal asphyxia, or their sex distribution (Table S3). However, newborn weights were significantly lower in infants born to mothers with mid-PD (2.99 ± 0.42 kg vs. 3.02 ± 0.43 kg, *P* = 0.027) or PPD (2.99 ± 0.53 kg vs. 3.01 ± 0.43 kg, *P* = 0.0228), with this decrease in weight specifically observed in female newborns (2.94 ± 0.42 kg vs. 2.97 ± 0.42 kg, *P* = 0.0127) but not in males (*P* > 0.05) (Table S4).

Mothers with early PD (K6 ≥ 9) (Table 1) had significantly higher K6 scores during mid-gestation (12.09 ± 3.25 vs. 2.41 ± 2.36) and higher EPDS scores at one month postpartum (11.65 ± 3.16 vs. 3.93 ± 2.22) compared to mothers without early PD (*P* < 0.0001). Similarly, mothers with mid-PD (K6 ≥ 9) had significantly higher early gestational K6 scores (12.72 ± 3.81 vs. 2.57 ± 2.44) and EPDS scores after one month postpartum (11.65 ± 3.16 vs. 3.93 ± 2.22) compared with those without mid-PD (*P* < 0.0001). After delivery, mothers with PPD (EPDS ≥ 9) exhibited significantly higher K6 scores during early and mid-gestation (12.72 ± 3.82 vs. 2.57 ± 2.44 and 12.10 ± 3.26 vs. 2.41 ± 2.36, respectively) compared to mothers without PPD (*P* < 0.0001). Additionally, mothers with early- (30.55 ± 4.93 vs. 31.83 ± 4.72), mid-PD (30.24 ± 4.90 vs. 31.81 ± 4.73) or PPD (29.83 ± 4.64 vs. 31.22 ± 4.72) (*P* < 0.0001) were significantly younger than those of controls, which is consistent with our previous report [37]. Across all time points (early gestation, mid-gestation, and postpartum), K6 and EPDS scores showed significant positive correlations with each other (R = 0.427–0.663, *P* < 0.001). No significant differences in K6 or EPDS scores or in maternal age at delivery were observed between primiparous and multiparous mothers.

Mann-Whitney *U* tests revealed that toddlers of mothers with PD (K6 or EPDS score ≥ 9) exhibited significantly higher TABS scores at all perinatal time points (*P* < 0.05; Table 1 and Table S5). In contrast, this association was not retained in univariate OLS regression analyses adjusted for maternal psychiatric history, antipsychotic medication use, socioeconomic status, developmental disorders, and child sex. Notably, the regression model identified a significant interaction between mid-pregnancy K6 and toddler sex (*P* = 0.031; Table S5). The discrepancy between the unadjusted non-parametric and adjusted parametric results is likely attributable to the study’s unbalanced design, in which the control group was 6- to 9-fold larger than the distress group. Under such conditions, non-parametric tests are less sensitive to sample size imbalance and thus provided a more reliable complement to our data. Therefore, Mann–Whitney *U* tests were employed in subsequent analyses.

The TABS scores of toddlers were significantly positively correlated with maternal early-K6 (R = 0.143; *P* < 0.001) and mid-K6 (R = 0.152; *P* < 0.001) scores (Table 1). Toddlers born to women with early-PD had significantly higher TABS scores (4.80 ± 3.20; n = 1,665) compared to those born to mothers without early-PD (3.88 ± 2.88; n = 9,745) (*P* < 0.0001, Table 1). Similarly, toddlers of mothers with mid-PD had significantly higher TABS scores (4.83 ± 3.16; n = 1,145) compared to toddlers of mothers without mid-PD (3.92 ± 2.91; n = 10,217) (*P* < 0.0001, Table 1). Furthermore, when using a cut-off value of K6 and EPDS scores at < 9 and ≥ 9, toddlers’ TABS showed a significant positive correlation with K6 or EPDS scores, regardless of whether their mothers had PD or PPD (R = 0.06―0.135, *P* < 0.05, Table 1). However, sex differences were observed in the correlation between K6 and TABS scores. Specifically, female toddlers born to mothers with PD during mid-gestation exhibited a significantly positive correlation (R = 0.102, *P* < 0.05), whereas no significance was observed in male toddlers (R = 0.057, *P* > 0.05).

Based on the OLS regression analyses of maternal distress and toddlers’ TABS scores (Table S5), both early- and mid-pregnancy K6 scores were positively associated with TABS. The regression coefficient for early pregnancy distress (β = 0.003, *P* = 0.009) was slightly greater than that for mid-pregnancy (β = 0.002, *P* = 0.047). To formally test whether early pregnancy exerted a stronger influence, we calculated the difference as β₍Early₎ − β₍Mid₎, yielding *z* = 0.71 (*P* = 0.48). This result indicates no statistically significant difference between the two gestational periods in their associations with toddlers’ TABS scores.

Using a TABS cut-off score of ≥ 15 [49], toddlers of women with early PD exhibited a 3.52-fold higher risk of developing autistic traits (odds ratio [OR]: 3.521, confidence interval [95% CI]: 1.278–9.700, *P* = 0.015). Similarly, toddlers of women with mid-PD showed a 3.98-fold higher risk (OR: 3.975, 95% CI: 1.222–12.929, *P* = 0.022). A significant positive correlation was also observed between EPDS and toddler TABS scores (R = 0.180; *P* < 0.001). Among mothers with PPD, their toddlers exhibited significantly higher TABS scores (4.91 ± 3.22; n = 3,200) compared to those of mothers without PPD (4.05 ± 2.95; n = 20,018) (*P* < 0.0001, Table 1), with a 2.56-fold increased risk of developing ART (OR: 2.564, 95% CI: 1.062–6.192, *P* = 0.036). Furthermore, among the mothers with PD (K6 ≥ 9) or PPD (EPDS ≥ 9), significantly positive correlations were observed between TABS scores and early K6 (R = 0.060, *P* < 0.05), mid-K6 (R = 0.080, *P* < 0.01), and EPDS (R = 0.131, *P* < 0.0001) scores (Table 1). Furthermore, among toddlers with TABS scores ≥ 15, none had mothers with a history of mental and neurodevelopmental disorders, particularly ASD (Table S6).

To investigate whether MPD is associated with sex differences in the male-biased prevalence of ASD, we analyzed TABS scores separately for male and female toddlers. Male toddlers exhibited significantly higher TABS scores than female toddlers, regardless of their mothers’ prenatal depression status (Table 1), consistent with previous studies; female toddlers exhibited fewer autistic characteristics than males [52]. Although male toddlers have a higher risk of ART with higher TABS scores, maternal PD or PPD had no significant effects on male toddlers’ ART diagnoses compared to male toddlers born to mothers without early PD (OR: 2.541, 95% CI: 0.656–9.844, *P* = 0.177), mid-PD (OR: 0.953, 95% CI: 0.121–7.534, *P* = 0.964), or PPD (OR: 2.042, 95% CI: 0.671–6.218, *P* = 0.209). In contrast, female toddlers born to mothers with early PD, mid-PD, or PPD had significantly higher rates of ART diagnoses compared to female toddlers of mothers without early PD (OR: 5.805, 95% CI: 1.170–28.813, *P* = 0.031), mid-PD (OR: 9.367, 95% CI: 1.886–46.528, *P* = 0.006), or PPD (OR: 5.470, 95% CI: 1.102–27.134, *P* = 0.038). In summary, early and mid-gestational K6 scores were strongly associated with EPDS, and both maternal PD and PPD were linked to a higher risk of ART in toddlers, with statistical significance specifically confirmed among female toddlers.

A previous study reported an association between maternal depressive symptoms and impaired maternal-fetal bonding during pregnancy and the postpartum period [53]. In this study, we further investigated the correlations between maternal bonding at one month postpartum, as measured by MIBS scores, and maternal psychological distress, as assessed by the K6 and EPDS scores (<9 or ≥9). As described above, MIBS showed positive correlations with K6, EPDS, and TABS (Table S2). This correlation remained significant (*P* < 0.0001) regardless of the presence or absence of early PD (K6 < 9: R = 0.173; K6 ≥ 9: R = 0.101), mid-PD (K6 < 9: R = 0.210; K6 ≥ 9: R = 0.106), or PPD (EPDS < 9: R = 0.167; EPDS ≥ 9: R = 0.100), as classified by K6 or EPDS scores (Table 1). However, among toddlers born to mothers with PPD, a significant positive correlation between MIBS and TABS scores was observed in female toddlers (R = 0.107, *P* < 0.0001) but not in male toddlers (R = 0.050, *P* > 0.05). In summary, female toddlers born to mothers with depression exhibited lower birth weight and a higher risk of ART development (TABS ≥ 15). Moreover, their TABS scores were positively correlated with maternal mid-gestational K6 and EPDS scores, as well as with impaired mother–infant bonding after delivery.

### CUMS induced depression-like behaviors in maternal mice and decreased body weight of newborns

To determine the pathogenic mechanism, we established a mice model of MPD. After CUMS exposure during pregnancy, the depressive-like behaviors of dams and the body weights of newborns were measured from P0 to P5. On P5, the dams were euthanized following the nest building test evaluation (Fig. 1a). Our results showed that CUMS exposure from G12 to P0 significantly reduced the body weight of dams (CUMS-dams) at P0 (*P* < 0.05; Fig. 1b) and resulted in lower body weight in their newborns (*P* < 0.001) (*F*_1,98_ = 66.28, *P* < 0.0001; Fig. 1c), without affecting litter size (*F*_8,98_ = 1.957, *P* = 0.06) compared to non-stressed dams. For evaluated depressive-like behaviors, the percentage of immobile time in the tail suspension test (P3) (*P* < 0.05, Fig. 1d) and the forced swim test (P4) (*P* < 0.001, Fig. 1e) were significantly increased in CUMS-dams in comparison with non-stressed controls but unchanged in serum corticosterone (P5) (*P* = 0.996, Fig. 1f). Meanwhile, CUMS-dams exhibited lower nest scores (P1 to P5) (*P* < 0.05, Fig. 1g) and significantly increased spending time for retrieving pups (P5) (*P* < 0.05, Fig. 1h). However, there were no differences on either total distance (*P* = 0.622, Fig. 1i) and time spent in the center zone (*P* = 0.787, Fig. 1j) in the CUMS-dams in comparison with non-stressed dams in the open-field test (OFT) (P2). Furthermore, there were no differences in percentage of sucrose intake (P0 to P3) (*P* = 0.977, Fig. 1k).

### The effects of prenatal stress on behavioral changes in juvenile mice

**Fig. 2:**
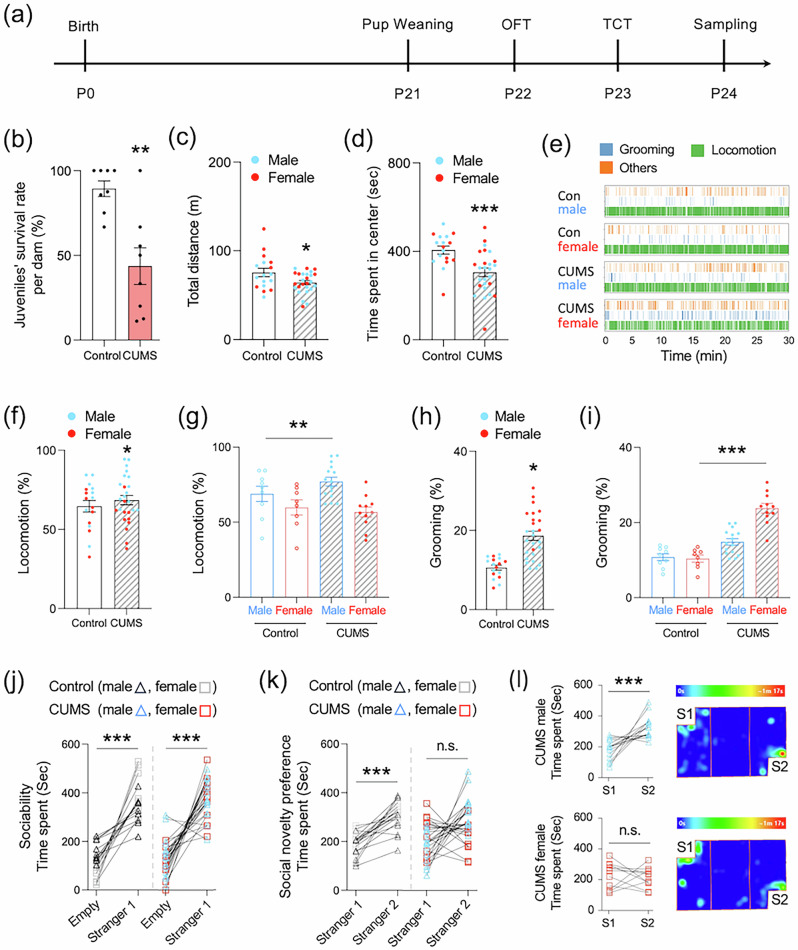
The effects of prenatal stress on behavioral changes in juveniles. **a** Schedule of behavioral experiments. Offspring were separated on postpartum day (P) 21 after weaning, the OFT and TCT were performed on P22 and P23, respectively. (**b**) The survival rates of juveniles at P21. The result of traveling total distance (**c**) and spending time in the center zone (**d**) of juveniles in the OFT. Bar graphs are presented as mean ± SEM. *P < 0.05. **P < 0.01. ***P < 0.001. **e** Visualization of behavioral state timelines in the OFT, showing periods of self-grooming (blue), locomotion (green), and other behaviors (orange). The results of sex differences in locomotion (**f,** **g**) and grooming behaviors (**h,** **i**) in the OFT of juveniles. Bar graphs are presented as mean ± SEM. *vs. Control (f. h), *P* < 0.05. ** vs. Male controls (**g**), *P* < 0.01. *** vs. female controls (**i**), *P* < 0.001. Tukey-adjusted post-hoc tests. (**j,** **k**) Social behaviors in juvenile offspring. Sociability was assessed by comparing the time spent interacting with a stranger mouse (Stranger 1) vs. an empty chamber (Empty) (**j**). Social novelty preference was assessed by comparing interaction time with the first unfamiliar mouse (Stranger 1) vs. a novel stranger (Stranger 2) (**k**). **l** Sex differences in social novelty preference (Stranger 1 vs. Stranger 2) and representative heatmaps in CUMS-juveniles, shown separately for males (top) and females (bottom). S1, Stranger 1. S2, Stranger 2. Data are presented as mean ± SEM. ***P < 0.001. n.s., not significant. Paired *t*-test. Control: males (black triangle), n = 9; females (grey square), n = 8. CUMS: males (blue triangle), n = 15; females (red square), n = 11. CUMS, chronic unpredictable mild stress. OFT, open-field test. TCT, three-chamber test.

Both control and CUMS-exposed dams were primiparous, which differs from the human cohort. Mice offspring were weaned and group-housed on P21 (juveniles) [54, 55], and the OFT and the three-chamber test (TCT) were performed at P22 and P23, respectively (Fig. 2a). Juveniles from CUMS-dams showed significantly lower survival rates on P21 (*P* < 0.01, Fig. 2b). The linear mixed-effects models (LMM) including dam as a random intercept revealed negligible between-litter variance for both total distance traveled ( ≈ 0.12 of total variance) and time spent in the center zone ( ≈ 0.13 of total variance) in the OFT, indicating minimal clustering by litter. Fixed effects revealed a significant reduction in total distance (*F*₁,₃₈ = 4.89, *P* = 0.032; Fig. 2c) and time spent in the center zone (*F*₁,₃₈ = 12.89, *P* = 0.0009; Fig. 2d) in juveniles born to CUMS dams, with no main effects of sex or CUMS × sex interaction (total distance: *F*₁,₃₈ = 0.87, *P* = 0.35; center zone: *F*₁,₃₈ = 3.12, *P* = 0.085). These results indicate that maternal stress reduced locomotor activity and increased anxiety-like behavior independently of sex. To further characterize behavioral phenotypes, hyper-locomotion and self-grooming during the 30-min OFT were quantified using *DeepLabCut*. A linear mixed-effects analysis revealed significant main effects of CUMS (*F*₁,₂₈ = 8.91, *P* = 0.005), sex (*F*₁,₂₈ = 12.34, *P* = 0.001), and a CUMS × sex interaction (*F*₁,₂₈ = 4.23, *P* < 0.05) on locomotion ( > 0.02 m/sec). CUMS-exposed males showed a significant increase ( + 12.7%, 95% CI [1.9, 20.6], P < 0.01), whereas females did not (–3.8%, *P* = 0.62; Fig. 2e–g). For self-grooming, a commonly used index of repetitive behavior [56, 57], CUMS also exerted a significant main effect (*F*₁,₃₅ = 15.27, *P* = 0.0004), with sex (*F*₁,₃₅ = 9.62, *P* = 0.004) and CUMS × sex interaction (*F*₁,₃₅ = 8.94, *P* < 0.01). Grooming increased in females ( + 128.5%, 95% CI [89.2, 167.8], *P* < 0.001) but not in males ( + 36.8%, *P* = 0.12; Fig. 2e, h, and i) (Supplementary Video 1 and 2). Litter effects were not significant for either locomotion (*F*₁₃,₂₈ = 1.18, *P* = 0.32; partial η² = 0.05) or self-grooming (*F*₁₂,₃₅ = 1.45, *P* = 0.19; partial η² = 0.08). Together, these findings indicate sex-specific stress sensitivity, with locomotion enhanced in male juveniles and excessive grooming in females following CUMS exposure.

Previous studies have shown that prenatal stress and cross-fostering impair social behaviors in male rat offspring, with females unexamined [58]. We analyzed sociability (Empty vs. Stranger 1) and social novelty preference (Stranger 1 vs. Stranger 2) in juveniles using the LMM with *Paired* as a random intercept and CUMS, sex, dam, and their interactions as fixed effects. Sociability was significantly increased when mice were allowed to contact a stranger mouse (Stranger 1) compared with an empty chamber (*F*₁,₇₆ = 5.73, *P* = 0.0192), and this effect did not differ by CUMS, sex, or CUMS × sex interaction (Fig. 2j). In contrast, social novelty preference was significantly affected by CUMS (*F*₁,₂₄ = 14.2, *P* = 0.0009) and showed a significant CUMS × sex interaction (*F*₁,₂₄ = 12.4, *P* = 0.0017) (Fig. 2k). Post-hoc paired *t*-tests revealed that CUMS-exposed males showed a robust increase in interaction time with the novel stranger (Stranger 2) relative to Stranger 1 (mean +147.5 ± 102.9 s, *t*(14) = 5.55, *P* < 0.0001), whereas CUMS-exposed females did not display a preference change (mean –12.4 ± 77.3 s, *t*(10) = –0.53, *P* = 0.61) (Fig. 2l). Mixed-effects analysis indicated negligible between-litter variance ( ≈ 0.08 of total variance), confirming that differences reflected individual rather than litter effects. These findings indicate that CUMS-exposed females showed impaired recognition of social novelty, consistent with cohort data suggesting that female toddlers born to mothers with MPD have a higher risk of ART.

In addition, juveniles at P21 did not exhibit marble-burying, digging, or nestlet-shredding behaviors during a 30-minute session, precluding reliable evaluation of these behaviors.

### Prenatal stress reduced transcription of microglial *Oxt* in dams and prefrontal cortical *Oxtr* in juvenile mice

**Fig. 3:**
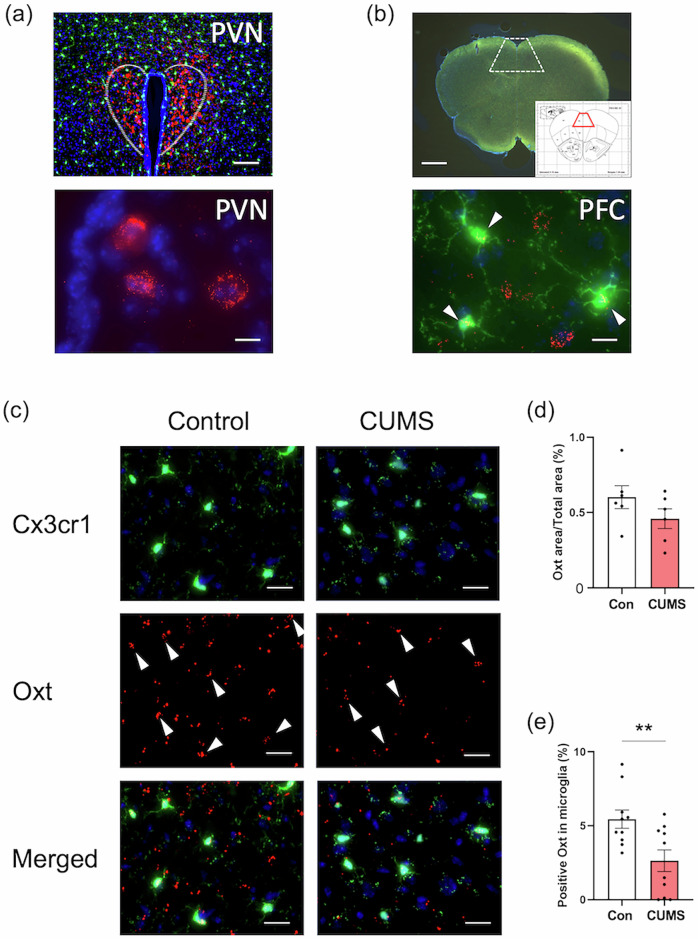
Prenatal stress reduced microglial *Oxt* transcription in the prefrontal cortex of dams. **a** *Oxt* transcription in the paraventricular nucleus (PVN) of the hypothalamus. Upper: The PVN region is enclosed by white dotted lines. Lower: *Oxt* transcripts and nuclei are shown in red and blue, respectively. Scale bars: 100 μm (upper; ×20), 10 μm (lower; ×100). **b** Microglial *Oxt* transcription in the prefrontal cortex (PFC). Upper: The PFC region is enclosed by white dotted lines, corresponding to the area outlined in red in The Mouse Brain in Stereotaxic Coordinates – *Second Edition* (DV: 2.46 mm relative to Bregma; 6.26 mm Interaural). Lower: CX3CR1 (microglia) and *Oxt* transcripts are shown in green and red, respectively. Scale bars: 1 mm (upper; ×5), 10 μm (lower; ×100). Arrows indicate microglia expressing *Oxt*. **c** CX3CR1 and *Oxt* are shown in green and red, respectively. Arrows indicate the microglial *Oxt*. Scale bars: 20 μm (×60). **d** was determined by calculating the density of the *Oxt* transcripts area/total area. **e** was determined by calculating the density of the positive *Oxt* signals in each microglia. Control, non-stressed dams; CUMS, chronic unpredictable mild stress exposed dams. Bar graphs are presented as mean ± SEM. **P < 0.01, vs. Control. Student’s *t*-test.

**Fig. 4:**
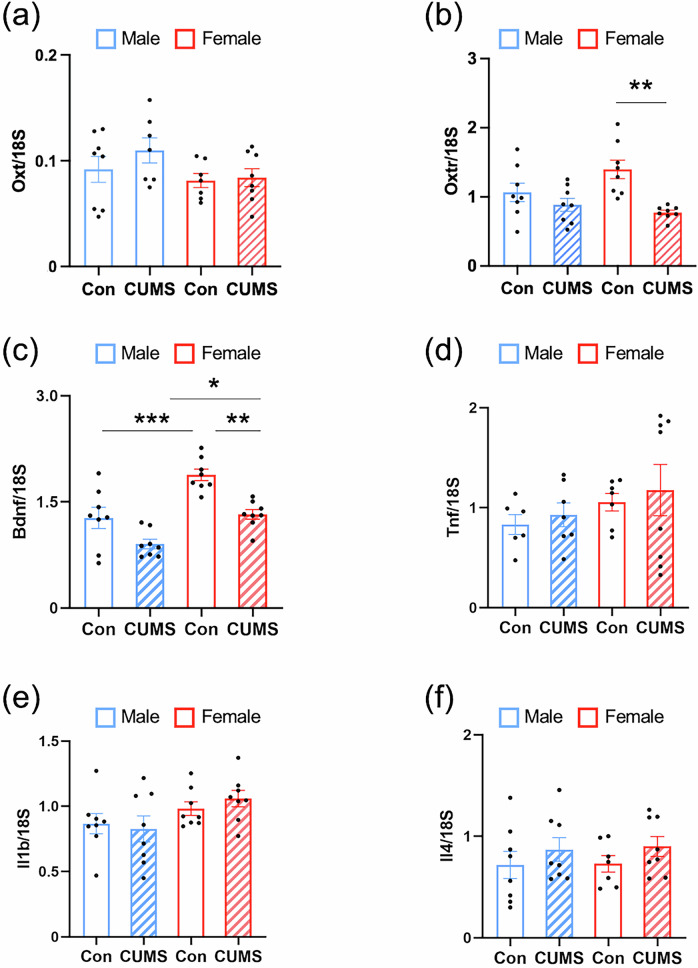
The transcription of *Oxt*, *Oxtr*, and cytokines in the prefrontal cortex of juveniles. **a** Levels of the mRNA encoding the *Oxt* relative to those of *18S*. **b** Levels of the mRNA encoding the *Oxtr* relative to those of *18S*. **c** Levels of the mRNA encoding the *Bdnf* relative to those of *18S*. **d** Levels of the mRNA encoding the *Tnf* relative to those of *18S*. **e** Levels of the mRNA encoding the *Il1b* relative to those of *18S*. **f** Levels of the mRNA encoding the *Il4* relative to those of *18S*. Bar graphs are presented as mean ± SEM. *P < 0.05, **P < 0.01, ***P < 0.001. One-way ANOVA with Bonferroni’s post-hoc. Con, offspring from non-stressed dams; CUMS, offspring from chronic unpredictable mild stress exposed dams. Control, Male, n = 8, Female, n = 8; CUMS, Male, n = 8, Female, n = 8.

Higher plasma OXT during pregnancy is associated with maternal bonding and attachment, suggesting its potential role in prepartum PPD prevention and mitigating its impact on mother–child relationships [22, 23]. Fluorescence in situ hybridization was used to assess the effects of maternal CUMS exposure on *Oxt* transcript expression in the dam’s brain, while RT-qPCR was used to determine *Oxt* and *Oxtr* transcript levels in the offspring’s PFC. Using in situ hybridization, we evaluated *Oxt* transcripts in the neurons of hypothalamic supraoptic nucleus (SON) and paraventricular nucleus (PVN) neurons (enclosed area by dotted lines in Fig. 3a) in brain of CUMS-dams on P5. There were no differences between CUMS-dams and their non-stressed controls in the PVN (*P* = 0.599, Fig. S2a and S2b) and SON (*P* = 0.678, Fig. S2c). The PFC is one of the key brain regions consistently implicated in mothers with PPD [59, 60] and maternal behavior across postpartum [61]. In CUMS dams, the total transcription of *Oxt* in the PFC was not statistically significant (*P* = 0.1845; Fig. 3b–d), nor was the total number of Cx3cr1-GFP⁺ microglia in the PFC (*P* > 0.05). However, microglial *Oxt* was significantly reduced in the PFC of CUMS-dams compared to that of non-stressed dams (*P* < 0.01, Fig. 3c and e). Furthermore, there were no significant differences in numbers of microglia of SON and PVN (*P* = 0.227, Fig. S2a and S2d). Based on behavioral sex differences in juveniles, RT-qPCR analysis was performed to assess the *Oxt* and *Oxtr* transcription in their PFC. Prefrontal cortical *Oxt* transcript levels did not differ significantly between CUMS-exposed and control groups (*F*_3,26_ = 1.939, *P* = 0.1481, Fig. 4a), whereas *Oxtr* transcript levels were significantly decreased in female juveniles born to CUMS-exposed dams (*P* < 0.01) (*F*_3,28_ = 6.707, *P* = 0.0015, Fig. 4b), consistent with previous postmortem brain reports of female patients with ASD [28].

### The effects of prenatal stress on transcriptions of cytokines in the PFC of dams and juvenile mice

Among the cytokines related to PPD in our previous report [10], no significant changes were observed in the transcriptional levels of inflammatory cytokines, including *Tnf* (*P* = 0.302, Fig. S3a) and *Il1b* (*P* = 0.997, Fig. S3b), or in the transcription of the anti-inflammatory cytokine *Il4* (*P* = 0.294, Fig. S3c) after delivery between CUMS-dams and their controls. The BDNF, a marker of microglial activation, its concentration after delivery was associated with the development of PPD [62] but did not change the transcription levels in the PFC of CUMS-exposed dams (*P* = 0.873, Fig. S3d). However, female *juveniles* born to CUMS-exposed dams exhibited significant decreased transcriptions of *Bdnf* (*P* < 0.01) (*F*_3, 28_ = 17.16, *P* < 0.001, Fig. 4c) but not males. There were no significantly changed transcriptions of cytokines in the PFC of juveniles from CUMS-exposed dams (*Tnf*: *F*_3, 24_ = 0.7709, *P* = 0.5216, Fig. 4d; *Il1b*: *F*_3, 28_ = 2.017, *P* = 0.134, Fig. 4e; *Il4*: *F*_3, 27_ = 0.734, *P* = 0.5411, Fig. 4f). In addition, the transcription of prefrontal cortical *Il10* was too low to be detected by RT-qPCR in both dams and their juveniles.

## Discussion

The cohort study found that maternal PD was positively associated with PPD development and correlated with higher TABS scores and poorer maternal bonding outcomes. Mothers with PD or PPD had a higher prevalence of toddlers exhibiting ART, with a notably higher incidence among female toddlers with lower birth weight. Regardless of maternal mental status, male toddlers exhibited significantly higher autistic-like characteristics than females, consistent with higher TABS scores in males driven by domains such as repetitive behaviors and speech/language concerns [63]. This pattern suggests that females may require greater risk exposure to exhibit comparable ART, potentially reflecting an innate protective mechanism [64]. Furthermore, female toddlers’ ART was positively associated with maternal mid-gestational PD, independent of family ASD history and antidepressant use after adjustment in OLS models (K6×sex interaction, *P* = 0.025). In mouse models, male juveniles born to CUMS-exposed dams exhibited increased locomotor activity, whereas females showed increased self-grooming and impaired social novelty preference—findings conceptually consistent with the cohort results. Collectively, these observations highlight the need for sex-specific norms for developmental assessments [52], as motor and global developmental delays appear to be more common in girls with ASD [65].

Mothers with antenatal depression have been reported to influence fetal neurodevelopment. Several studies indicate that antenatal depression is associated with altered expression of genes, such as *PEG3* [66], *HTR1A* and *NPY2R* [67] involved in placental nutrient transport and vascular development. Moreover, mothers with PPD experience impaired maternal-infant bonding, difficulties in breastfeeding, and poor maternal caregiving behaviors and responsiveness to infant cues [68]. Such disruptions in early-life social interactions, which are crucial for normal infant neurodevelopment, may further contribute to adverse developmental outcomes. Notably, early childhood is a period of rapid brain growth and neuroplasticity [69], and Zwaigenbaum et al. highlighted the early intervention is beneficial for children under age of three may mitigate the severity of emerging ART [70], which may also serve as a predictor of long-term outcomes in adulthood among individuals with ASD [48, 71, 72]. These mechanisms are hypothesized pathways and require replication in larger, well-controlled cohorts.

Low pregnant plasma OXT [22] and its dysregulation are considered critical for maternal behaviors and social bonding and may be disrupted during PPD, impairing social and emotional regulation associated with ASD [73–76]. In the current study, we observed reduced *Oxt* transcript levels in the PFC microglia of postpartum dams exposed to prenatal CUMS, while no significant changes in the SON and PVN. While these observations are consistent with microglial involvement, they do not establish causality, several studies determined the microglial dysfunctions in the patients with depression and murine models. For instance, patients with major depression during severe episodes exhibit microglial activation in the PFC [77]. Furthermore, positron emission tomography has shown that microglia increase in the anterior cingulate cortex during episodes of major depression [78]. In murine models, microglia also respond to prenatal stress; mice with microglial autophagy deficiency show higher inflammatory levels, further instigating PPD-related behaviors [79]. Moreover, CUMS-induced abnormal behaviors observed in the juveniles may not be attributed to immune dysregulation in the dams but rather directly linked to altered microglial activity (e.g., synaptic pruning [36], morphological changes [35], autophagy [40], and oxidative stress [33]), as a promising target for investigating the mechanisms underlying PPD.

The mechanism underlying the sex differences in toddlers’ ART related to MPD remains unclear. Our results demonstrated decreased *Oxtr* transcript levels in the PFC of female juveniles born to CUMS-exposed dams, partially consistent with clinical postmortem studies in females with ASD [28]. Furthermore, *OXTR* variant (*rs2254298*) in females has been associated with increased attachment anxiety [80], potentially contributing to impaired mother-infant bonding. However, it remains unknown how infants respond to persistently depressed maternal states that fail to meet attachment needs [81]. We also observed reduced *Bdnf* transcript levels in the PFC of female juveniles born to CUMS-exposed dams. The concurrent reductions of *Oxtr* and *Bdnf* in females may reflect coordinated developmental regulation; specifically, early-life decreases in both genes during female rodent development [17] could contribute to sex-differential vulnerability to maternal-stress–related neurodevelopmental dysfunction. However, this remains a hypothesis requiring direct testing (e.g., cell-type–specific co-expression analyses). Moreover, amygdala BDNF dysfunction reported in female patients with major depression and in stressed female mice [82] may further contribute to sex differences. Elucidating the OXTR–BDNF relationship in the PFC and its influence on social behavior may yield mechanistic insights and candidate biomarkers that help explain why current clinical trial evidence does not support routine OXT treatment for core ASD symptoms in children [25].

Notably, female offspring born to mothers with PPD have significantly lower bodyweight, which is consistent with our recent clinical report on mothers with PPD symptoms and lower-weight newborns, specifically in females [37]. These associations may be confounded by gestational age, obstetric complications, or neonatal factors; sensitivity analyses are needed. Several studies reported that MPD affects infant development and is related to sex. Magnetic resonance imaging studies have reported sex differences in the white matter of offspring exposed to prenatal stress, suggesting that female neonates may exhibit a unique developmental sensitivity or response to prenatal antidepressant exposure [83]. Although the association between ASD pathogenesis in female children is poorly evidenced, a previous study demonstrated that the risk of depression at age 18 following prenatal maternal exposure is higher in girls [84]. In maternally stressed mouse dams during pregnancy, the greater transport of corticosterone across the placenta in female offspring compared to males [85] may contribute to sex-differential developmental trajectories.

These preliminary observations underscore the importance of screening and supporting maternal mental health, as well as conducting longitudinal, preregistered studies that integrate broader behavioral assessments and mechanistic biomarkers (e.g., the OXTR–BDNF axis). Our results suggest that MPD, particularly during mid-pregnancy, may be more strongly associated with ART in female toddlers. More broadly, these findings highlight the critical role of maternal mental health in offspring neurodevelopment and support early, family-centered interventions to mitigate the impact of prenatal distress on maternal and child outcomes, especially mother–infant bonding.

### Limitation

This study had some limitations. First, the cohort sample size was relatively small; larger studies are needed to improve generalizability. As data were obtained from a single-region Japanese population, regional and cross-cultural differences were not considered, and validation in independent, diverse cohorts is required. Validation with independent cohorts from diverse regions is necessary. The human cohort included both primiparous and multiparous mothers, whereas all mouse dams were primiparous; although litter effects were controlled and multiple births excluded, parity-related influences on maternal behavior cannot be ruled out. Information on NICU stay (presence, absence, or duration) was also unavailable. In the murine model, we avoided strong prenatal stressors such as prolonged restraint, food restriction, or tail pinching, which increase stillbirths [86]. Other CUMS paradigms—before pregnancy or after postpartum—may better model distinct depressive phenotypes. Under the mild stress conditions used here, OFT showed no reduction in total distance, and center-zone exploration was comparable to previous reports [87]. Differences in strain, stress duration, and testing time likely contributed to variability. Despite the behavioral test battery, serum corticosterone, cytokines, and sucrose intake remained unchanged, possibly reflecting that postpartum affective symptoms differ from non-postpartum depression or anxiety [88]. Based on the need to better model human ASD, further optimization of prenatal stress paradigms and behavioral parameters in C57BL/6 J mice is warranted, including longitudinal assessments beyond 4 weeks due to the absence of marble-burying and nestlet-shredding behaviors at 3 weeks, as well as validation of decreased maternal microglial *Oxt* transcription using microglial OXT knockout or overexpression models.

## Supplementary information

Supplementary methods

Supplementary Tables

Supplementary Video 1

Supplementary Video 2

Select_random_mice.py

Dlc_behavior_timeline.py

Dlc_behavior_summary.py

## Data Availability

The cohort data are available through the Tohoku Medical Megabank database (https://dbtmm.megabank.tohoku.ac.jp/catalogue/3.1.1). All remaining data and supplementary information relevant to this study are included in this paper.
