# Supplementary material for: Sex differences in the risk of autistic-related traits in toddlers born to mothers with perinatal depression: Evidence from human cohort and mouse study
Source: Mol Psychiatry. 2026 Feb 4;31(6):3229–42. doi: 10.1038/s41380-026-03456-z (PMC13190272; doi:10.1038/s41380-026-03456-z)
Supplement: Supplementary file 1 — Supplementary methods [file 41380_2026_3456_MOESM1_ESM.docx]

**Sex Differences in the Risk of Autistic-Related Traits in Toddlers Born to Mothers with Perinatal Depression: Evidence from Human Cohort and Mouse Study**

**Supplementary figures:**

**
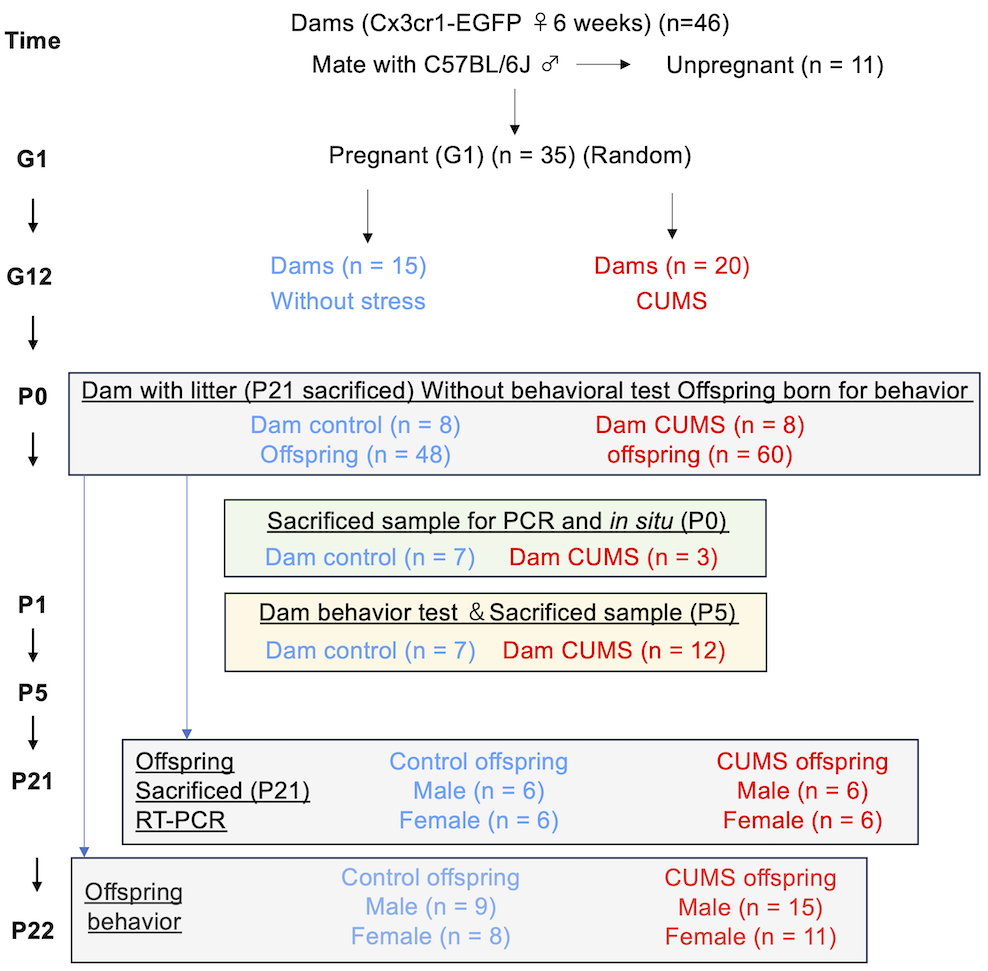
**

**Figure S1.** Selection of mice for ELISA, RT-qPCR, *in situ* hybridization, and behavioral testing. Selection of mice for ELISA, RT-qPCR, in situ hybridization, and behavioral testing. Sample size estimation was performed using G*Power (version 3.1.9.7; t test, two-tailed, α = 0.05, power = 0.8). Assuming an effect size of Cohen’s d = 1.4, the required number of offspring per sex per group was n = 6. To ensure sufficient pups, 15 control and 20 CUMS dams were prepared, considering possible pregnancy loss under stress. CUMS, chronic unpredictable mild stress.

**
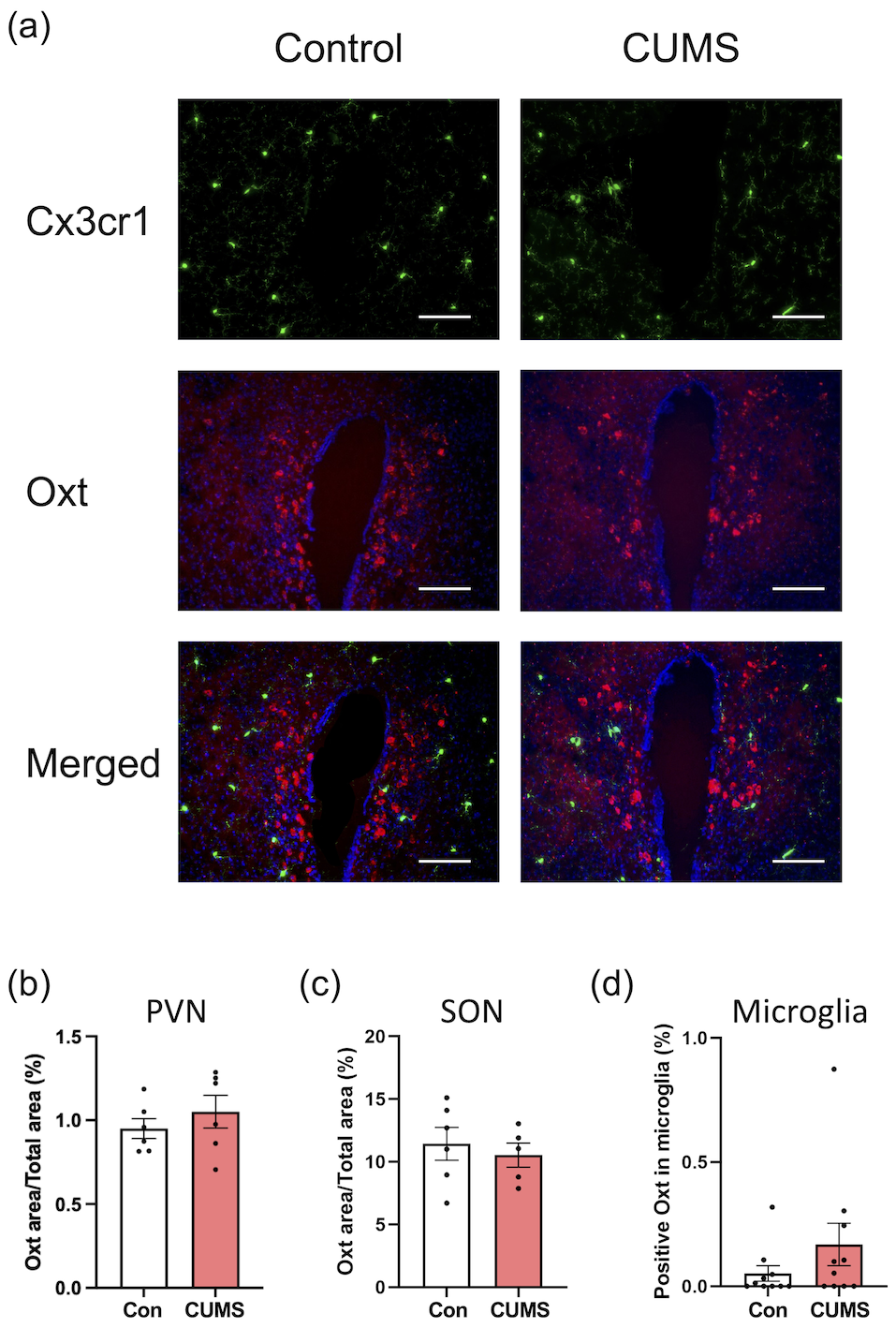
**

**Figure S2.** Transcriptions of microglial *Oxt* did not change in the supraoptic and paraventricular nuclei of the hypothalamus after prenatal stress. In the merged images in (a), CX3CR1 (microglia) and *Oxt* are shown in green and red in the hypothalamus, respectively. Scale bars, 50 μm (×60). Transcription of *Oxt* in paraventricular (b) and supraoptic (c) nuclei was respectively determined by calculating the density of the *Oxt* transcripts area/total area. (d) was determined by calculating the density of the positive *Oxt* signals in each microglia. Control, non-stressed dams; CUMS, chronic unpredictable mild stress exposed dams. Bar graphs are presented as mean ± SEM. Student’s *t*-test. SON, supraoptic nucleus of the hypothalamus. PVN, paraventricular nucleus of the hypothalamus.


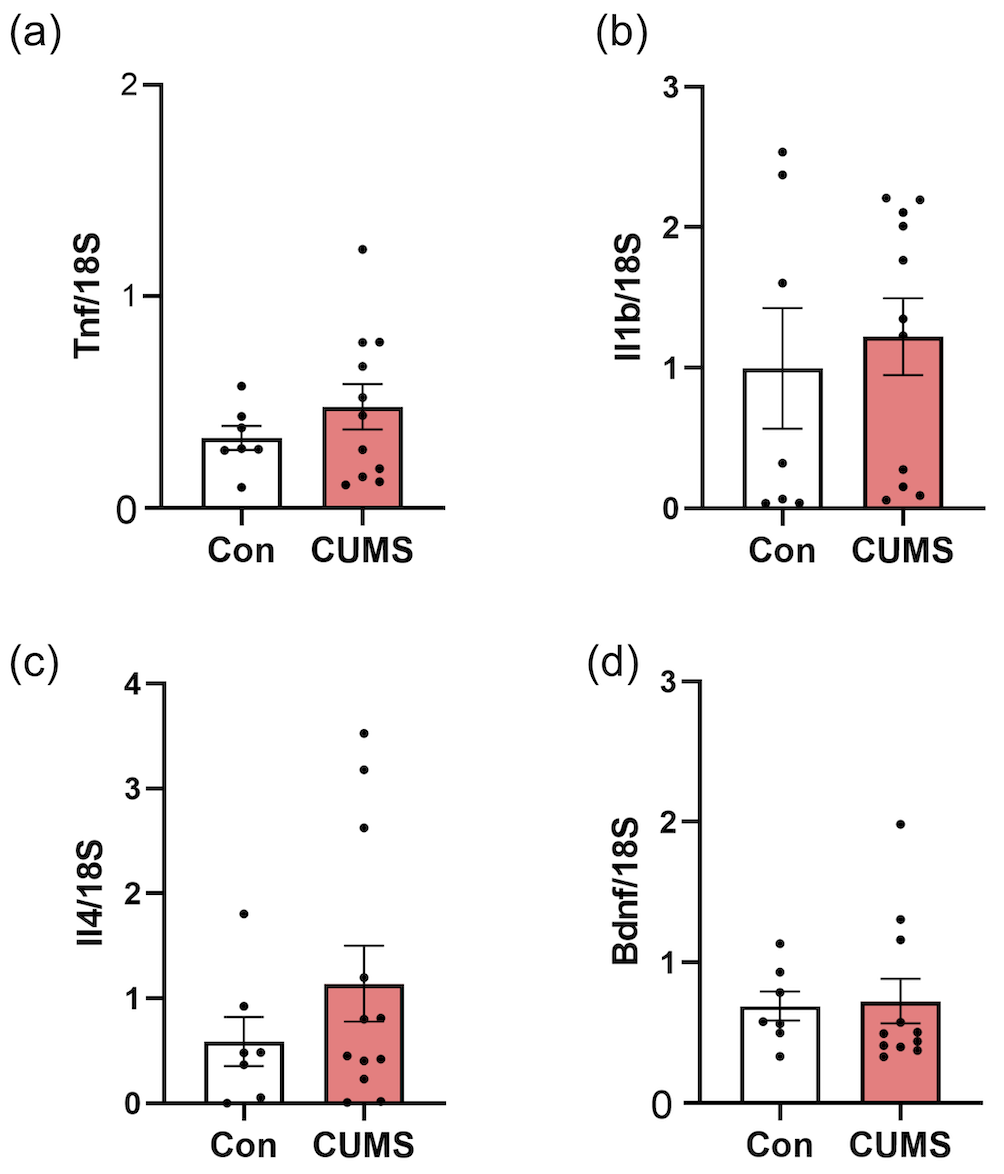


**Figure S3.** The effects of prenatal stress on transcriptions of cytokines in the maternal prefrontal cortex. (a) Levels of the mRNA encoding the *Tnf* relative to those of *18S*. (b) Levels of the mRNA encoding the *Il1b* relative to those of *18S*. (c) Levels of the mRNA encoding the *Il4* relative to those of *18S*. (d) Levels of the mRNA encoding the *Bdnf* relative to those of *18S*. Control, n = 7; CUMS, n = 12. Control, non-stressed dams; CUMS, chronic unpredictable mild stress exposed dams. Bar graphs are presented as mean ± SEM. Student’s *t*-test.

**
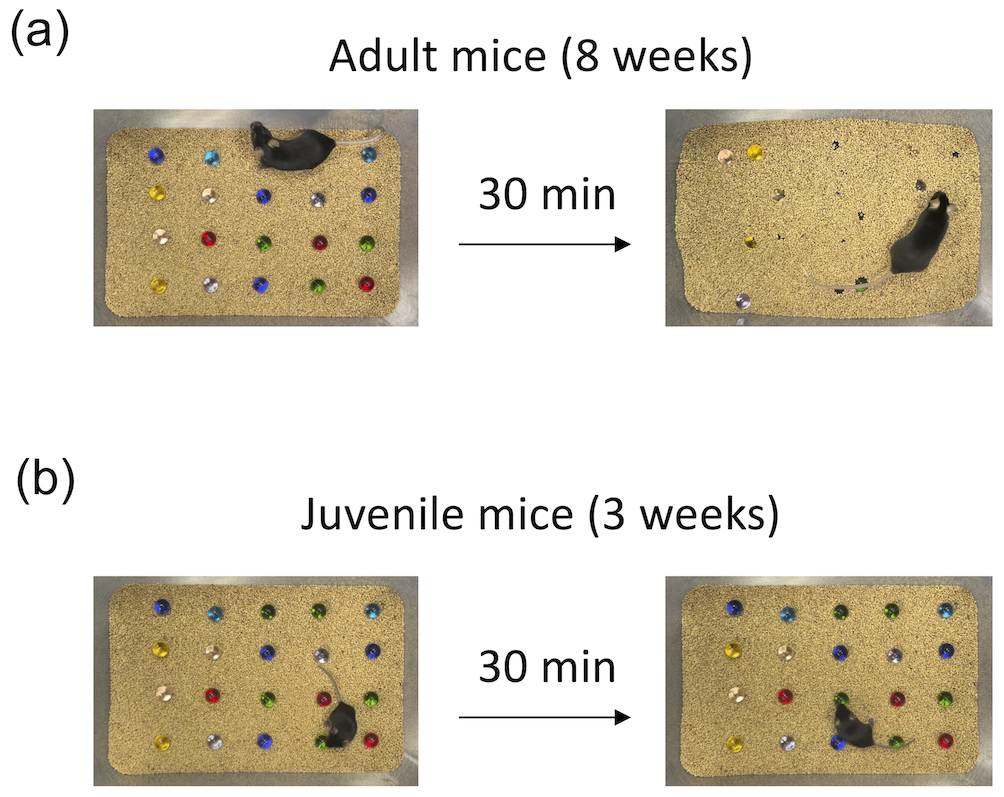
**

**Figure S4.** Marble-burying test. (a) Eight-week-old mice exhibited active digging and marble-burying behavior, burying 17 of 20 marbles after a 30-min test. Left, at the start, right, after 30 min. (b) Three-week-old mice showed no digging but rather avoidance behavior, and no marbles were buried after the 30-min test. Left, at the start, right, after 30 min.

**Supplementary Video 1**. Self-grooming behavior of a female juvenile mouse born to a non-stressed dam, with 16 body-part key points labeled using *DeepLabCut* (total 22 s).

**Supplementary Video 2**. Self-grooming behavior of a female juvenile mouse born to a CUMS-exposed dam, with 16 body-part key points labeled using *DeepLabCut* (total 2 min 43 s). CUMS, chronic unpredictable mild stress-exposed dams.

**Supplementary methods:**

*Human cohort study*

The sample data supporting the current study are available from the Tohoku Medical Megabank (TMM) Organization (https://www.megabank.tohoku.ac.jp/english/sample/), specifically from the TMM Birth and Three-Generation (TMM BirThree) Cohort Study (https://www.megabank.tohoku.ac.jp/english/research/cohortbiobank/birthree/). This analysis targeted women during pregnancy and childbirth enrolled in the TMM BirThree Cohort Study, a general population-based prospective cohort study that began in 2011. The study protocol was approved by the Ethics Committee of the Tohoku University Graduate School of Medicine (Approval No. *2024-1-677*). Women diagnosed with gestational diabetes mellitus were excluded. In the current study, all personal information from questionnaires, clinical data, and biospecimens was processed exclusively by the independent Anonymization Management Office of the TMM, where personal identifiers were replaced with unique study-specific codes.

*Animals*

The care, housing, procedures, and euthanasia of animals strictly complied with the Guidelines for the Care of Laboratory Animals of the Tohoku University Graduate School of Medicine (Sendai, Japan), the Act on Welfare and Management of Animals (Japan), the Fundamental Guidelines for Proper Conduct of Animal Experiments and Related Activities in Academic Research Institutions (MEXT, Japan), and the Guidelines for Proper Conduct of Animal Experiments (Science Council of Japan). The investigator was blinded to group allocation during the experiment and outcome assessment.

*Measurement of bodyweight, number of offspring, and offspring survival rate*

Body weights of dams and offspring were measured on P0. The number of offspring was recorded on the day of birth and again three weeks after weaning. The offspring survival rate was calculated as follows: Survival rate (%) = (number of offspring after weaning / number of offspring at birth) × 100%.

*Mouse selection for ELISA, RT-qPCR, in situ hybridization, and behavioral testing*

This experimental design was established to investigate the effects of maternal chronic unpredictable mild stress (CUMS) exposure on both maternal behavioral phenotypes and the molecular and behavioral outcomes of the offspring. At each stage, mice were randomly assigned using a custom Python script (Supplementary file: *select_random_mice.py*). A total of 46 six-week-old female CX3CR1^GFP/GFP^ mice (Fig. S1) were used in this study. These females were mated with C57BL/6J males, resulting in 35 pregnancies (G1), while 11 females did not become pregnant. Pregnant dams were randomly assigned to either an untreated control group (n = 15) or a CUMS group (n = 20).

After parturition (P0), subsets of dams were allocated for different experimental purposes. To avoid potential effects of behavioral testing on maternal care, some dams were euthanized at P21 without behavioral assessment for ELISA, RT-qPCR, and *in situ* hybridization experiments, and their offspring were subsequently used for RT-qPCR and behavioral experiments (Control: dams n = 8, offspring n = 48; CUMS: dams n = 8, offspring n = 60). At P0, a separate group of dams was euthanized for RT-qPCR analysis (Control: n = 7, CUMS: n = 3). At P5, another subset underwent behavioral testing prior to tissue collection (Control: n = 7, CUMS: n = 12). Offspring were divided into groups for molecular and behavioral analyses. At P21, one group was euthanized for RT-qPCR analysis (Control: males n = 6, females n = 6; CUMS: males n = 6, females n = 6). Another group underwent behavioral testing at P22 and P23 (Control: males n = 9, females n = 8; CUMS: males n = 15, females n = 11).

*Open field test (OFT)*

Anxiety-like behaviors were first determined by OFT. The OFT was performed in an arena (50×50×30 cm). A series of 10×10 cm zones was divided, the middle nine blocks were defined as the center zone (30×30 cm), and the outer zone consisted of 16 blocks other than the center zone^1^. The mice were placed in the center of the open field, recorded over 30 min by video, and analyzed using ANY-maze video tracking software Ver 6.1 (Stoelting Co., Wood Dale, IL). The parameters studied were total distance and amount of time spent in the center squares^2^.

*Sucrose preference test (SPT)*

Each SPT (after pregnancy and birth) was performed over two consecutive days. Each mouse was habituated to drinking water from two special bottles that consisted of a tube and stopper fitted with ballpoint sippers (Ancare, Bellmore, NY, USA). In the first 24 h, the mice were given two 25 ml bottles of 1% sucrose solution. In the next 24 h and during the test period, the mice were given free choice between a bottle of tap water and a bottle of 1% sucrose solution, and the bottle positions were switched every 12 h. The test period was 24 h; after 24 h, the consumption of both sucrose and water was measured. The sucrose preference rate was calculated as follows: SPT (%) = sucrose intake (g)/ [water intake (g) + sucrose intake (g)] × 100.

*Tail suspension test (TST)*

Each mouse was suspended by the tail using a masking tape placed < 1 cm from the tip of the tail. The behaviors of the mice during the test were recorded using a camera for 6 min. Immobility time was manually calculated using videos. The results were expressed using the immobility index [immobility index = immobile time (Sec) / total time (Sec) × 100]^3^.

*Forced swim test (FST)*

The mice were placed in a transparent plastic cylinder (25×11 cm) filled with water (room temperature) 15 cm high^4^. The procedures were recorded on a video for 6 min. Immobility time was manually calculated using videos. The results were expressed using the immobility index [Immobility index = immobile time (Sec) / total time (Sec) × 100].

*Nest building test (NBT)*

The quality of nest construction after delivery was evaluated^5^. Briefly, Enviro-dri^®^ nesting material (Shepherd, Cleveland, OH, USA) was placed on the cage floor of each pregnant mouse at G19. Nest building was scored from P1 to P5 for five consecutive days between 9:00 and 11:00 a.m., based on the volume of nesting material and the nest’s length, width, and height. The nesting score was defined as follows: undisturbed nesting material (score 0), disturbed nesting material (score 1), flat nest (score 2), cup (score 3), incomplete dome (score 4), and complete dome (score 5). In addition, the integrity of the nest wall was evaluated in quarters, and 0.25 points were added for each intact quarter of the wall. Higher scores indicated higher-quality nests and lower depression-like behaviors.

*Pup retrieval test (PRT)*

At the beginning of the test^5^, the dam was removed from the breeding cage, and three randomly selected pups were moved from the nest to the other three corners. The dam was then reintroduced to the cage and allowed to move freely under recording video for 10 minutes. The time spent retrieving all three pups was recorded; however, if the dam did not retrieve all pups within 10 minutes, the time was recorded as 10 minutes.

*Three-chamber test (TCT)*

At P21, juvenile offspring^6^ were weaned to group housing, and their sociability and preference for social novelty were determined by TCT at P23 (Fig. 2a)^7^. The apparatus (51 × 34 × 22cm) consisted of three equal-sized chambers (17 × 34 cm), including a central chamber and two side chambers with doors (7 cm × 5 cm), allowing the experimental mouse to move freely between the three chambers. Small cages were installed to isolate one mouse in each chamber, enabling olfactory communication but preventing complete physical contact. At the beginning of the test, each mouse was allowed to acclimate to the environment for five minutes. After a 5-minute session, the test mouse was placed in the central chamber of the apparatus, and the unfamiliar mouse (Stranger 1, sociability) was placed in a small cage in one of the side chambers. An empty small cage was placed in the other side chamber, and the test mouse was allowed to explore the entire apparatus for ten minutes. A second unfamiliar mouse (Stranger 2, preference for social novelty) was then caged and placed in the previously empty cage. The test mouse was allowed ten minutes to assess its preference for social novelty, reflecting recognition and motivation for interaction with a novel conspecific. A video camera recorded the duration of each chamber and later analyzed it using ANY-Maze video tracking software. After TCT behavioral testing, juvenile mice were randomly selected (n = 8) using an Excel random number generator (=RAND), and their prefrontal cortex was isolated for RT-qPCR analyses.

*Self-grooming behavior of juvenile mice*

The data of OFT at P22 of juvenile mice that wean from 21 days (Fig. 2a) were used for grooming analyses^8, 9^, 30-fps (1,920*1,080) MP4 files prepared with Wondershare Filmora (Wondershare Technology, Shenzhen, China) while retaining the original video properties were analyzed using *DeepLabCut* (v2.3.11 or v3.0.0rc10)^10, 11^ in a Python (v3.12.6) environment with TensorFlow and PyTorch 2.2.2 on macOS Sequoia 15.6.1. Python was further used to process the training results and to analyze the HDF5 (.h5) files, a hierarchical data format that enables efficient storage and organization of large-scale datasets containing x- and y-axis coordinate data. Each mouse was labeled with 16 body points (1. Nose, 2. Left_eye, 3. Right_eye, 4. Left_forelimb, 5. Right_forelimb, 6. Left_hindlimb, 7. Right_hindlimb, 8. Left_ear, 9. Right_ear, 10. Neck, 11. Body_anterior, 12. Body_middle, 13. Body_posterior, 14. Tail_base, 15. Tail_middle, and 16. Tail_tip) (Supplementary Videos). Body parts that were not visible in a given frame were not labeled. Thirty frames were extracted from each 30-min video and used for training with the following parameters: display iterations = 5,000, number of snapshots = 15, maximum epochs = 1,000, and save frequency = every 200 epochs, generating .h5 files for subsequent analysis. The optimized performance was assessed using *DeepLabCut*’s “Evaluate Network” analysis on a held-out .h5 dataset, confirming strong generalization prior to model deployment for further analyses. Grooming results were summarized using a custom Python script (Supplementary File: *dlc_behavior_summary.py*) to calculate the duration of self-grooming, locomotion, and stationary behaviors (hypo-locomotion or rest). Locomotion was defined as framewise body displacement exceeding a velocity threshold of V_MOVE = 8.0 px/s, corresponding to sustained translational movement across the arena. Stationary behavior was defined as body displacement below this threshold, provided that the posture did not satisfy criteria for grooming. Self-grooming was identified by low body displacement (V_GROOM < 3.0 px/s) in combination with characteristic postural features, specifically a shortened nose–ear distance (< 15 px) indicative of head and forelimb movements directed toward the body. Tail-base coordinates were used as auxiliary markers to enhance discrimination of grooming bouts. To minimize misclassification due to transient fluctuations, behavioral states shorter than 30 consecutive frames (~1 s at 30 fps) were merged with the adjacent state. The behavioral data from juvenile mice were visualized as timeline graphs using a custom Python script (Supplementary File: *dlc_behavior_timeline.py*).

*Marble-burying test and Nestlet Shredding test*

Juvenile mice were tested in a standard cage (19 × 29 × 13 cm) containing 4 cm of walnut shell bedding (RP-752, Vivaria, Osaka, Japan) for the marble-burying test^12^. Twenty glass marbles (Ø 1.5 cm; 4.5 g) were arranged in a 4 × 5 grid on the bedding surface. Each juvenile mouse was placed in the corner of the cage for 30 min under 50 lux illumination. The number of marbles buried (≥ 2/3 surface covered) was recorded as the primary measure. Video scoring provided latency to dig and total digging time as secondary outcomes. Litter was modeled as a covariate in the analyses.

The Nestlet Shredding test^12^ was performed by placing a single mouse in a novel cage containing a pre-weighed nestlet. The mouse was left undisturbed for 30 min, with food and water withheld during the test period. Following the test, the mouse was returned to its home cage. The remaining unshredded nestlet material was collected, dried overnight, and weighed to determine the percentage shredded. This measure quantified repetitive shredding behavior. These behaviors were first examined independently in 3-week-old C57BL/6J male and female juveniles; however, no digging, marble-burying (Fig. S4), or nestlet-shredding behavior (e.g., <5% nestlet weight loss, no visible tearing) was observed during the 30-min tests, suggesting limited expression of these behaviors at this early juvenile stage, consistent with previous reports indicating reduced stability of behavioral phenotypes in young mice^13^.

*Tissue and serum collection*

After the behavioral tests, conducted 24 h later between 09:00 and 11:00 h, mice were euthanized by decapitation, and their brains were immediately removed and placed on ice. The frontal lobe was dissected and stored at –80 °C. Blood was collected into sterile 2-ml polypropylene tubes (Cat. no. 0030120094. Eppendorf, Hamburg, Germany), and serum was isolated by centrifugation (15,000 g, 4 °C, 10 min) and stored at –80 °C.

*Real-time qPCR*

Total RNA was extracted from the PFC using the RNeasy Mini Kit with on-column DNase treatment to eliminate genomic DNA contamination (Cat. no. 74104. Qiagen, Valencia, CA, USA) and subsequently reverse-transcribed into cDNA using random primers and the SuperScript VILO cDNA Synthesis Kit (Cat no. 11754050 Invitrogen, Carlsbad, CA, USA). Quantitative PCR was performed with gene-specific primers and iQ SYBR Green Supermix (Cat. no. 170-8882. Bio-Rad, Hercules, CA, USA) in a total reaction volume of 20 μL, consisting of 10 μL of 2× iQ SYBR Green Supermix, 0.8 μL of each primer, 2 μL of cDNA template, and nuclease-free water. Amplification conditions were as follows: initial denaturation at 95°C for 3 min, followed by 60 cycles of 95°C for 10 s and 60°C for 30 s. Reactions were run in triplicate, and fluorescence was measured at each cycle to determine threshold cycle (Ct) values. Relative transcript levels were calculated using the comparative Ct (*Δ*Ct) method, normalized to the S18 reference gene. 18S rRNA was used as the internal control to normalize the results. The forward and reverse primers for *18S* (amplicon size: 87 bp) were 5’- GTAACCCGTTGAACCCCATT -3’ and 5’- CCATCCAATCGGTAGTAGCG -3’, respectively. The forward and reverse primers for *Oxt* (amplicon size: 161 bp) were 5’-TTCTTCGTGCAGATGTGGAG-3’ and 5’-CCTTCAGGTACCGAGCAGAG-3’, respectively. The forward and reverse primers for *Oxtr* (amplicon size: 187 bp) were 5’-AGCCCCCAGTCTGTATCCTT-3’ and 5’-CTCCCTTTGCAGAACTCAGG-3’, respectively. The forward and reverse primers for *Tnf* (amplicon size: 157 bp) were 5’-TCGTAGCAAACCACCAAGTG-3’ and　5’-CCTTGTCCCTTGAAGAGAACC-3’, respectively. The forward and reverse primers for *Bdnf* (amplicon size: 242 bp) were 5’-aggacagcaaagccacaatg-3’ and 5'-gtgctcaaaagtgtcagcca-3’, respectively. The forward and reverse primers for *Il1b* (amplicon size: 230 bp) were 5’-GCCCATCCTCTGTGACTCAT-3’ and 5-AGGCCACAGGTATTTTGTCG-3’, respectively. The forward and reverse primers for *Il4* (amplicon size: 177 bp) were 5'-TCAACCCCCAGCTAGTTGTC-3’ and 5'-TGTTCTTCGTTGCTGTGAGG-3’, respectively. The forward and reverse primers for *Il10* (amplicon size: 191 bp) were 5'-GGTTGCCAAGCCTTATCGGA-3’ and 5'-ACCTGCTCCACTGCCTTGCT-3’, respectively. Several quality control measures were implemented to ensure assay specificity and accuracy. Primers were designed using Primer 3 to avoid off-target effects and ensure specificity. Negative (no-template control) and positive controls were included in each run to detect contamination and non-specific amplification, respectively. A post-qPCR melting curve analysis was performed to verify the specificity of amplification products. Only single specific peaks were observed, confirming the primer specificity. The efficiency of each primer pair was validated by constructing standard curves using serial dilutions of cDNA. The R^2 values of standard curves were consistently greater than 0.99, indicating a high degree of correlation and reliability. Multiple replicate experiments were performed on the same samples to ensure reproducibility of amplification results. Undetected samples were excluded from statistical analyses. All real-time qPCR experiments were performed in accordance with the internationally recognized MIQE guidelines^14^.

*Corticosterone assay*

A corticosterone ELISA kit (Cat. no. ADI-900-097. Enzo Life Sciences Inc, PA, USA) was used to detect the serum corticosterone levels of dams on P5 after behavioral tests, as per the manufacturer’s instructions. Absorption was recorded at 405 nm using a Spectra MAX M2e microplate reader (Molecular Devices, Sunnyvale, CA, USA). For quality control, each sample was assayed in duplicate. The intra-assay coefficient of variation (CV) for all samples was consistently below 5%. Any sample showing a CV above 5% was excluded from the analysis.

*Fluorescence in situ hybridization (FISH)*

Mice were anesthetized using a medetomidine-midazolam-butorphanol mixture (medetomidine 0.3 mg/kg, Kyoritsu Seiyaku, Tokyo, Japan; midazolam 4 mg/kg, Maruishi Pharmaceutical Co., Ltd., Osaka, Japan; butorphanol tartrate 5 mg/kg, Meiji Animal Health Co., Ltd., Tokyo, Japan) and transcardially perfused with phosphate-buffered saline (162-19321. FUJIFILM Wako Pure Chemical Corp. Osaka, Japan), followed by a 4% paraformaldehyde phosphate buffer solution (161-20141. FUJIFILM Wako). To determine the expression of Oxt in microglia, brains were immersed in 4% paraformaldehyde for 24 h and then transferred to 30% sucrose for 24 h. After the brains were rapidly frozen in OCT compound (Sakura Finetek, Torrance, CA, USA), coronal brain sequential 10 μm sections were made using a LEICA cryostat (CM3050; Leica Biosystems, Nussloch, Germany) and mounted on slides. Two consecutive slides were used for FISH and microglial detection. The ViewRNA Tissue Assay Fluorescence Kit (QVT0800, Invitrogen, Scientific, Waltham, MA, USA) was used for FISH as per the manufacturer’s instructions. In detail, the slides were heated in a chamber at 95.5℃ for 10 minutes. After washing the slides with phosphate-buffered saline (PBS), the working protease solution was added, followed by a labeled *Oxt* mouse probe (VB1-3031107-VC LabelAlexa Fluor 546/594, Invitrogen) hybridized at 40℃ for 1 h. After washing with wash buffer, the secondary antibody (ViewRNATM Tissue Alexa FluorTm 594 Type 1 Module; Invitrogen) was incubated at 40℃ for 30 min to perform the amplification step. Nuclei present in the slices were stained using an Autofluorescence Quenching Kit with DAPI (SP-8500-15; Vector Laboratories, Burlingame, CA, USA) to diminish unwanted autofluorescence. Cell images were acquired using a fluorescence microscope (Axio Scope.A1; Carl Zeiss, Oberkochen, Germany). The levels of *Oxt* mRNA and Cx3cr1 in the PVN were determined using the ImageJ 1.53 K software (NIH Image, Bethesda, MD, USA). Furthermore, the fluorescent image of microglia from CX3CR1^GFP/+^, which was taken sequentially, was combined with FISH-imaged *Oxt* to obtain a merged image of *Oxt* transcripts in microglia.

*Statistical analysis*

Cohort study:

Variables [the Kessler Psychological Distress Scale–6 items (K6), the Edinburgh Postnatal Depression Scale (EPDS), the Tokyo Autistic Behavior Scale (TABS), and the Mother-to-Infant Bonding Scale (MIBS) scores] were analyzed as continuous measures (e.g., correlations), and the results were consistent with those obtained using categorical cutoffs. A categorical approach (K6 or EPDS < 9 vs. ≥ 9) was also applied, as these thresholds have recognized clinical relevance and enable comparison with our previous studies^15-17^.

In detail, the association between maternal perinatal psychological distress and offspring TABS scores was primarily examined using non-parametric Mann–Whitney *U* tests, which are appropriate for data with unequal group sizes and heteroscedasticity. To further address potential confounding and effect modification, we additionally performed univariate Ordinary Least Squares (OLS) linear regression models to assess crude associations. Maternal distress was treated as a binary exposure variable (K6 or EPDS score < 9 vs. ≥ 9), and the models were adjusted for maternal psychiatric history, smoking and drinking habits, educational attainment, household income, use of antipsychotic medication, and child sex. An interaction term between the K6 group and child sex was also included (Table S7). Model diagnostics indicated a violation of the homoscedasticity assumption (Levene’s test, *P* < .001) and highly unbalanced group sizes. In mothers with a history of psychiatric depression or anxiety, the use of antipsychotic medication, SSRIs (escitalopram, sertraline, fluvoxamine), another antidepressant (mirtazapine), hypnotics (zolpidem, suvorexant), and benzodiazepine or related anxiolytics/hypnotics (ethyl loflazepate, etizolam, flunitrazepam, clotiazepam, lorazepam) was also analyzed as covariates. Results are presented as unstandardized regression coefficients with their standard deviation (SD), 95% confidence intervals (CI), and p-values. All analyses were performed using Python 3.9.5 (*pandas 2.2.2*, *statsmodels 0.14.0*).

*Assessment of gestational sensitive period*

To determine whether maternal psychological distress during early or mid-pregnancy exerted differential effects on toddlers’ autistic-related traits, two complementary analyses were conducted. First, we compared the unstandardized coefficients obtained from separate univariate OLS regression models, in which the TABS score was regressed on maternal K6 score measured either in early or mid-pregnancy, adjusted for the same set of covariates across models. The difference between the two regression coefficients was evaluated using a z-test for independent estimates:

$$z=\frac{\beta_{Early}-\beta_{Mid}}{\sqrt{SE_{Early}^{2}+SE_{Mid}^{2}}}.$$

Second, we conceptually examined a joint model including both Early-K6 and Mid-K6 as simultaneous predictors of TABS, while controlling for the moderate correlation between the two measures (r = 0.66; Table S2). This approach allows assessment of the relative contribution of each gestational period while accounting for shared variance between predictors. All analyses were two-tailed, and statistical significance was defined as *P* < 0.05.

Animal experiments:

Behavioral and *in situ* hybridization outcomes of dams were analyzed using Student’s *t*-test between CUMS and control groups. Behavioral and RT-qPCR outcomes in juveniles (P23) were analyzed for the effects of maternal stress (CUMS vs. control), sex (male vs. female), and their interaction. Because multiple juveniles were derived from the same dam, observations were not statistically independent. To account for this hierarchical structure, linear mixed-effects models (LMMs) were fitted with CUMS, sex, and their interaction (CUMS×sex) as fixed effects and dam as a random intercept (*mixedlm()* function in statsmodels.formula.api). To account for the non-independence of offspring within the same litter, we fitted a linear mixed model (LMM) with litter (dam) specified as a random intercept, following *Lazic & Essioux* (2013)^18^ and *Golub* et al. (2020)^19^. Between-litter (σ²_dam) and residual (σ²_residual) variance components were extracted to evaluate the proportion of behavioral variability attributable to the litter level, conceptually equivalent to σ²_dam / (σ²_dam + σ²_residual). A small litter-level variance indicates that behavioral variability was largely explained at the individual rather than the litter level. As a sensitivity analysis, OLS models with cluster-robust standard errors by dam were also fitted (*ols()* in statsmodels.formula.api with get_robustcov_results(cov_type="cluster", groups=...)), providing litter-adjusted inference without explicit random-effects modeling. A two-way analysis of variance (ANOVA) was conducted on paired behavioral data from the TCT, with CUMS and sex as fixed factors, using the *anova_lm*() function from the statsmodels.stats.anova library. All analyses were performed in Python (statsmodels package, version 0.14.0) or GraphPad Prism version 10 (GraphPad Software, Inc., San Diego, CA, USA). Two-sided *P* values < 0.05 were considered statistically significant. Data are presented as mean ± SEM.

**Reference**

1. Seibenhener ML, Wooten MC. Use of the Open Field Maze to measure locomotor and anxiety-like behavior in mice. *J Vis Exp* 2015; (96)**:** e52434.

2. Moura CA, Oliveira MC, Costa LF, Tiago PRF, Holanda VAD, Lima RH *et al.* Prenatal restraint stress impairs recognition memory in adult male and female offspring. *Acta Neuropsychiatr* 2020**:** 1-6.

3. Ueno H, Takahashi Y, Murakami S, Wani K, Matsumoto Y, Okamoto M *et al.* Effect of simultaneous testing of two mice in the tail suspension test and forced swim test. *Sci Rep* 2022; **12**(1)**:** 9224.

4. Can A, Dao DT, Arad M, Terrillion CE, Piantadosi SC, Gould TD. The mouse forced swim test. *J Vis Exp* 2012; (59)**:** e3638.

5. Seki T, Yamagata H, Uchida S, Kobayashi A, Watanabe Y, Nakagawa S. A novel mouse model of postpartum depression using emotional stress as evaluated by nesting behavior. *Sci Rep* 2021; **11**(1)**:** 22615.

6. Verhoeven D, Perry S, Pryharski K. Control of influenza infection is impaired by diminished interferon-γ secretion by CD4 T cells in the lungs of toddler mice. *J Leukoc Biol* 2016; **100**(1)**:** 203-212.

7. Moy SS, Nadler JJ, Perez A, Barbaro RP, Johns JM, Magnuson TR *et al.* Sociability and preference for social novelty in five inbred strains: an approach to assess autistic-like behavior in mice. *Genes Brain Behav* 2004; **3**(5)**:** 287-302.

8. Geuther BQ, Peer A, He H, Sabnis G, Philip VM, Kumar V. Action detection using a neural network elucidates the genetics of mouse grooming behavior. *Elife* 2021; **10**.

9. Peça J, Feliciano C, Ting JT, Wang W, Wells MF, Venkatraman TN *et al.* Shank3 mutant mice display autistic-like behaviours and striatal dysfunction. *Nature* 2011; **472**(7344)**:** 437-442.

10. Mathis A, Mamidanna P, Cury KM, Abe T, Murthy VN, Mathis MW *et al.* DeepLabCut: markerless pose estimation of user-defined body parts with deep learning. *Nat Neurosci* 2018; **21**(9)**:** 1281-1289.

11. Nath T, Mathis A, Chen AC, Patel A, Bethge M, Mathis MW. Using DeepLabCut for 3D markerless pose estimation across species and behaviors. *Nat Protoc* 2019; **14**(7)**:** 2152-2176.

12. Angoa-Pérez M, Kane MJ, Briggs DI, Francescutti DM, Kuhn DM. Marble burying and nestlet shredding as tests of repetitive, compulsive-like behaviors in mice. *J Vis Exp* 2013; (82)**:** 50978.

13. Menashe N, Salama Y, Steinauer ML, Spaan JM. Do behavioral test scores represent repeatable phenotypes of female mice? *J Pharmacol Toxicol Methods* 2022; **115:** 107170.

14. Bustin SA, Benes V, Garson JA, Hellemans J, Huggett J, Kubista M *et al.* The MIQE guidelines: minimum information for publication of quantitative real-time PCR experiments. *Clin Chem* 2009; **55**(4)**:** 611-622.

15. Kikuchi S, Murakami K, Obara T, Ishikuro M, Ueno F, Noda A *et al.* One-year trajectories of postpartum depressive symptoms and associated psychosocial factors: findings from the Tohoku Medical Megabank Project Birth and Three-Generation Cohort Study. *J Affect Disord* 2021; **295:** 632-638.

16. Yu Z, Matsukawa N, Saigusa D, Motoike IN, Ono C, Okamura Y *et al.* Plasma metabolic disturbances during pregnancy and postpartum in women with depression. *iScience* 2022; **25**(12)**:** 105666.

17. Ono CT, Yu Z, Obara T, Ishikuro M, Murakami K, Kikuya M *et al.* Association between low levels of anti-inflammatory cytokines during pregnancy and postpartum depression. *Psychiatry Clin Neurosci* 2023; **77**(8)**:** 434-441.

18. Lazic SE, Essioux L. Improving basic and translational science by accounting for litter-to-litter variation in animal models. *BMC Neurosci* 2013; **14:** 37.

19. Golub MS, Sobin CA. Statistical modeling with litter as a random effect in mixed models to manage "intralitter likeness". *Neurotoxicol Teratol* 2020; **77:** 106841.
